# Protocol for a feasibility randomised controlled trial of Screening and Enhanced Risk management for Vascular Event-related Decline in Memory (SERVED Memory)

**DOI:** 10.1136/bmjopen-2017-017416

**Published:** 2017-11-28

**Authors:** Phyo Kyaw Myint, Yoon K Loke, William Davison, Katharina Mattishent, George Christopher Fox, Robert Fleetcroft, David Turner, Lee Shepstone, John F Potter

**Affiliations:** 1 Institute of Applied Health Sciences, University of Aberdeen, Aberdeen, UK; 2 Norwich Medical School, University of East Anglia, Norwich, UK

**Keywords:** stroke, dementia, vascular medicine, stroke medicine

## Abstract

**Introduction:**

Stroke is a leading cause of death and disability. The development of dementia after stroke is common. Vascular risk factors (VRF) which contribute to stroke risk can also contribute to cognitive decline, especially in vascular dementia (VaD). There is no established treatment for VaD, therefore strategies for prevention could have major health resource implications. This study was designed to assess whether patients with early cognitive decline after stroke/transient ischaemic attack (TIA) can be easily identified and whether target-driven VRF management can prevent progression to dementia.

**Objectives:**

The primary objective is to establish the feasibility of recruitment and retention of patients with early cognitive decline to a randomised controlled trial of enhanced VRF management. Secondary objectives include: (a) to determine the potential clinical benefit of the intervention; (b) to estimate the sample size for a future definitive multicentre randomised controlled trial; (c) to inform a future economic evaluation; (d) to explore the link between VRF control and the incidence of cognitive impairment on longitudinal follow-up in a UK population after stroke/TIA with current routine management.

**Methods:**

100 patients with cognitive decline poststroke/TIA will be recruited from stroke services at the Norfolk and Norwich University Hospital. After collection of baseline data, they will be randomised to intervention (3 monthly follow-up with enhanced management) or control (treatment as usual by the general practitioner). At 12 months outcomes (repeat cognitive testing, VRF assessment) will be assessed. A further 100 patients without cognitive decline will be recruited to a parallel observational group from the same site. At 12 months they will have repeat cognitive testing.

**Ethics and dissemination:**

Ethical approval has been granted in England. Dissemination is planned via publication in peer-reviewed medical journals and presentation at relevant conferences.

**Trial registration number:**

42688361; Pre-results.

**Strengths and limitations of this study**

- The protocol uses a validated cognitive screening test, which is sensitive and specific for the detection of mild cognitive impairment as well as dementia.
- Data will be collected on a range of vascular risk factors.
- The study is open-label, but repeat cognitive testing will be completed by a member of the research team who is blinded to allocation and baseline cognitive status.
- The chosen follow-up period of 12 months may limit our ability to detect changes in cognition.

## Introduction

### Background

Stroke is one of the leading causes of death and disability^1^ and current demographic trends suggest that the total numbers of people with a stroke will rise due to the ageing population.^2^ Cognitive decline after stroke poses a significant problem considering that up to 30% of patients may potentially develop dementia as early as 3 months after their cerebrovascular event.^3^ Stroke may unmask previously unrecognised cognitive impairment,^4 5^ or may trigger new cognitive decline due to VaD, Alzheimer’s disease or mixed pathology.^6 7^

The World Alzheimer Report emphasised the benefit of early diagnosis with future savings from delayed institutionalisation and care costs across the disease course.^8^ Similarly, the UK Government has identified the timely diagnosis of dementia in primary care as a priority.^9^ An effective strategy in preventing VaD could have major resource implications, with at least one in five dementia cases having a VaD element and dementia costing the UK economy £23 billion per year.^10^ Most importantly, ‘*how best to improve cognition after stroke’* was reported to be the highest priority research topic in a survey of patients with stroke.^11^ Identifying patients who have signs of early cognitive decline after stroke or TIA could provide a window of opportunity for saving resources and improving patient outcomes if further cognitive decline could be prevented.^6^

It is reported that dementia is common after stroke and TIA.^3 4 12 13^ Reported rates range from 7.4% up to 41.3% with the variance mostly dependent on the mix of the cohort (eg, rates are higher in secondary care cohorts and those with higher rates of recurrent stroke).^14^ Our previous work shows that the risk of developing cognitive impairment appears to be greater in people with higher numbers of VRF^4^ and other work suggests that the presence of cardiovascular risk factors increases the risk of early cognitive decline progressing to dementia.^15^ Improved control of VRF leading to enhanced secondary stroke prevention may therefore help to prevent further cognitive decline after stroke/TIA in high-risk patients with evidence of early cognitive impairment.

### Rationale for the study

Observational evidence indicates that both VaD and Alzheimer’s dementia may have risk factors in common with stroke, namely VRF such as high blood pressure (BP) and diabetes.^16 17^ Despite this, whether intervening to control these risk factors can prevent dementia remains unclear.^16 18^ First, trials of antihypertensive therapy have been inconsistent. However, they may have been limited by high rates of treatment in placebo groups, high dropout rates and short follow-up.^18^ Of note, a large trial which recruited patients with stroke/TIA (PROGRESS) did demonstrate reduced cognitive decline, but not dementia, with treatment.^16 18^ Furthermore, meta-analysis of placebo-controlled trials suggests that antihypertensive therapy reduces the risk of dementia.^19^ Second, two randomised controlled trials have assessed the use of statins and found no benefit on cognition despite reduction in cholesterol levels.^20^ Third, in the ADVANCE study intensive blood glucose control in type 2 diabetics successfully reduced microvascular complications, but did not reduce rates of dementia.^18^ Finally, whether anticoagulation for atrial fibrillation (AF) can prevent cognitive decline is, at present, not addressed by the available evidence.^16 17^ In spite of this uncertainty there is evidence, as alluded to earlier, that recurrent stroke is an important factor in poststroke dementia.^14^ Given that treating VRF is beneficial for secondary stroke prevention, it therefore remains plausible that this could also have an impact on cognitive decline poststroke and further research is justified. Evidence to support this comes from a randomised controlled trial in Germany, which demonstrated a significant reduction in the need for long-term care in older adults following an intervention involving systematic identification and evidence-based treatment of cardiovascular risk factors.^21^ Although two trials similar to ours have investigated the use of an intervention targeted at controlling VRF for preventing cognitive decline after stroke and neither demonstrated a benefit of intervention at 12 months,^22 23^ a key difference with our study is that we will be targeting patients who already have signs of cognitive impairment at baseline and therefore are at higher risk of further decline.

Routine cognitive testing using validated measures which are simple and quick, such as the Montreal Cognitive Assessment (MoCA), which are shown to be sensitive and specific in detecting vascular-related cognition can identify those who are at risk of developing decline.^24–26^ We believe therefore, that detection of early cognitive decline in stroke and TIA is feasible at the time of diagnosis in secondary care and we propose that enhanced (target driven) VRF control is clinically effective, cost-effective and safe.

The reported incidence of dementia poststroke is variable, with the highest rates being over 40%.^7 14^ Reporting differences are strongly attributable to variation in the cohorts studied.^14^ There is also some evidence to suggest that cognitive decline after TIA or minor stroke (defined as National Institute of Health Stroke Scale <3) may be transient.^27^ However, there is a lack of data based on the current UK population. This study will therefore incorporate a parallel observational arm with a view to generating relevant epidemiological data in order to provide better insight regarding cognition after stroke/TIA in this patient population.

### Study objectives

The primary objective of the study is to determine the feasibility of randomising patients who have signs of early cognitive decline, but no dementia, into routine risk factor management or enhanced risk factor management by their GP, and to assess adherence to the proposed intervention by enrolled participants.

The secondary objectives are as follows:

- Determine the potential clinical benefit of enhanced control of VRF in preventing progression of cognitive decline and the development of dementia poststroke/TIA.
- Assess indicative cost-effectiveness of this intervention.
- Estimate the sample size for a future definitive multicentre randomised controlled trial.
- Identify any adverse events due to the intervention, including rates of recurrent stroke/TIA.
- Explore the incidence of cognitive impairment on longitudinal follow-up in a UK population after stroke/TIA with current routine risk factor management.

## Methods and analysis

### Study overview

This study is a single-centre, open-label parallel group study to determine the feasibility of conducting a randomised controlled trial in a National Health Service (NHS) setting on patients following stroke or TIA who have early cognitive decline. The aim is to target risk factors more intensively through enhanced monitoring and control of VRF compared with usual care. We wish to estimate the potential clinical impact and cost-effectiveness of this intervention, and the sample size for a future multicentre definitive study in an NHS setting.

**Figure 1:**
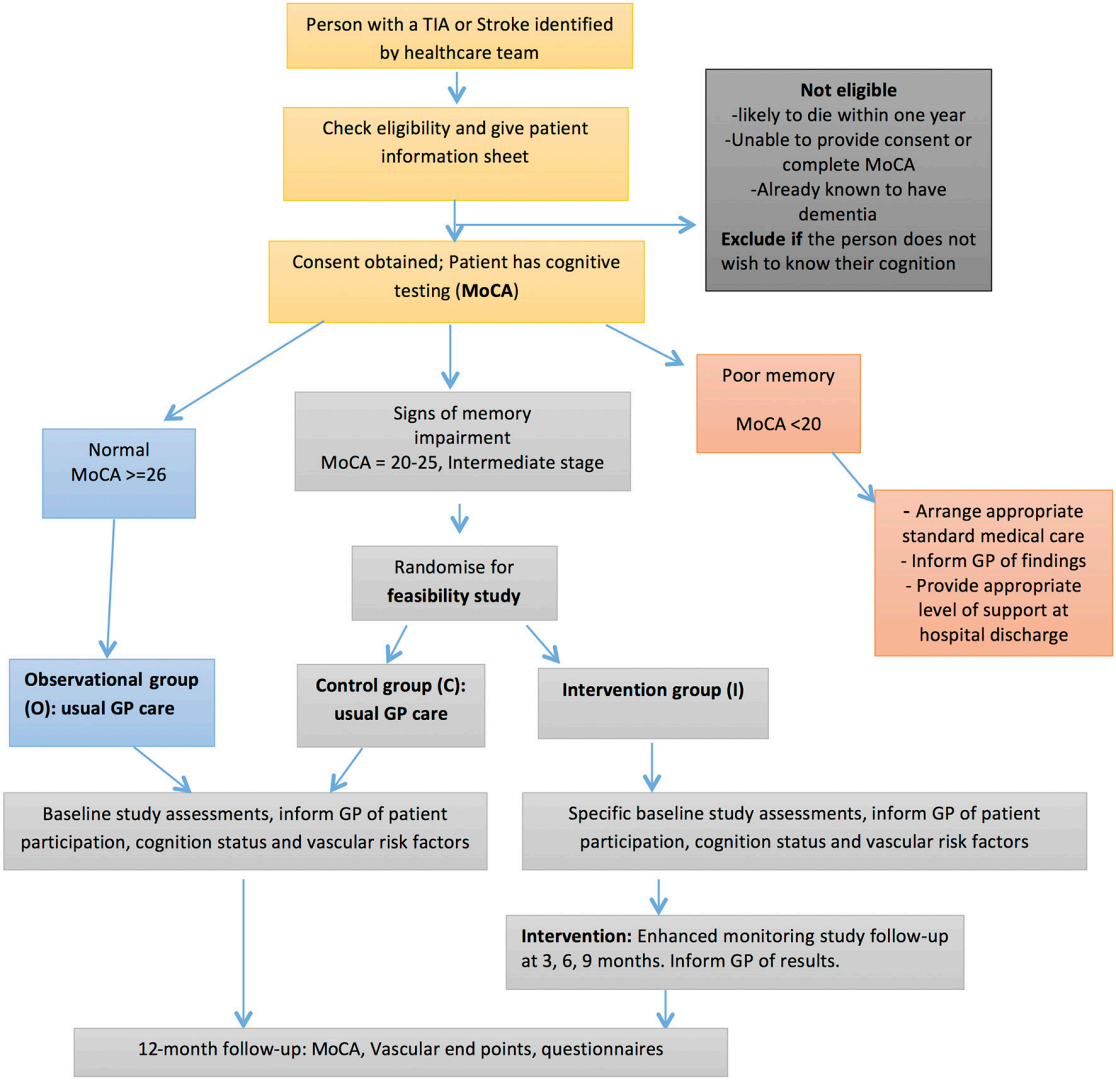
Summary of the study design depicting the flow of participants through the study. Steps detailed include the identification and recruitment of participants, allocation and randomisation into the study arms based on Montreal Cognitive Assessment (MoCA) score, and the timing of intervention and follow-up visits. GP, general practitioner; TIA, transient ischaemic attack.

There is a parallel observational study arm for patients with no evidence of cognitive decline who will have their VRF and cognitive function assessed at follow-up. The objective of the parallel observational study is to better understand the link between VRF, their control and the development of cognitive decline after a cerebrovascular event. Combining the control arm of the feasibility trial and observational cohort will provide further information on these links, including a realistic estimate of the magnitude of effect of enhanced risk factor management in planning a future trial. A summary of the study design is provided in figure 1. Recruitment commenced in November 2015, was completed in July 2017 and final follow-up data collection will be in July 2018. The study has been registered on 16 April 2015: International Standard Randomised Controlled Trial Number 42688361.

### Trial participants

All adult patients with confirmed stroke (first/recurrent) or TIA, identified within 8 weeks of diagnosis will be considered for the trial.

#### Inclusion criteria

- Participant is willing and able to give informed consent for participation.
- Male or female, aged 18 years or above.
- Diagnosed clinically and radiologically with stroke (infarct or haemorrhage) or TIA.

#### Exclusion criteria

The participant may not enter the trial if ANY of the following apply:

- Established dementia.
- Life expectancy <1 year.
- Comorbidities that adversely affect their ability to accurately complete the MoCA.
- Patients who do not wish to know about their cognition.

### Identification of participants

Eligible patients will be given a detailed Patient Information Sheet (PIS) and Consent Form for consideration. After 24 hours the study team will contact the patient again and those who agree to participate will provide written informed consent and undergo a simple and validated cognitive screening test (MoCA), unless this has already been carried out by the clinical team as part of their routine care, whereupon that score can be used for study purposes. If a MoCA has been administered by the TIA clinic team the patient will be given a PIS in clinic and be followed up by phone to discuss entry into the study. Patients who have previously attended stroke services may be screened retrospectively from the Capture TIA/Stroke hospital database and followed up by phone. Those who have a MoCA score ≥26 and who verbally consent to be contacted about the study are sent a study summary sheet, a study invitation letter and consent form. If they wish to participate, the completed consent form will be returned and countersigned by a delegated member of the study team. Those who do not have a MoCA score following their stroke or TIA are invited for an appointment to give consent to take part in the study and carry out the MoCA. The patient will be enrolled into the appropriate arm of the study depending on the MoCA score.

### Assessing capacity and obtaining informed consent

The participant must personally sign and date the latest approved version of the Informed Consent Form. This will then be countersigned by a delegated member of the research team before any trial-specific procedures are performed. Written and verbal versions of the PIS will be presented to the participants detailing the trial rationale; participant involvement and responsibilities; the implications and constraints of the protocol; safeguards and processing of blood tests. It will be clearly stated that the participant is free to withdraw from the trial at any time for any reason without prejudice to future care, and with no obligation to give the reason for withdrawal. The member of the research team who takes consent will be delegated to do so, be familiar with the study and be suitably qualified to obtain consent for research purposes. A copy of the signed informed consent will be given to the participant, and another will be stored at the Norfolk and Norwich University Hospital Clinical Research Trials Unit (CRTU). The original signed form will be retained in the patient’s medical records.

### Allocation into study arms

Allocation is based on the patient’s MoCA score, interpreted in the following manner:

- Score ≥26 indicates normal cognition, with this score being chosen to maximise the sensitivity of the test for detecting early cognitive decline.
- Score 20–25 suggests early cognitive decline.^24 25^
- Score ≤17 suggests possibility of dementia.^28^

### Normal cognition (MoCA score ≥26)

Patients with normal cognition will be informed of their result and will continue to receive usual care by their clinicians. They will be asked to confirm their willingness to continue to participate in the observational study (group O) and will be followed up at 12 months.

### Intermediate stage (MoCA score 20–25)

Patients will be informed of their result and asked to confirm their willingness to continue to participate in the feasibility trial. They will be randomised into one of two groups: control arm (group C) or intervention arm (group I). Randomisation will be based on computer-generated blocked randomisation managed by the Norwich CRTU.

Patients in group C will receive usual care by their clinicians and will be followed up at 12 months.

Patients in group I will undergo enhanced VRF management through assessment by the study team at 3, 6 and 9 months. Specific aims will be set for each modifiable risk factor and the GP will be informed about these targets and the results of each visit.

### Greater degree of cognitive decline (MoCA score <20)

In view of the greater extent of cognitive impairment continued participation in the study is not suitable for these patients. They will be referred to specialist services where relevant, and their GP will be informed.

### Interventions to be measured

#### Blood pressure

Existing evidence demonstrates that lowering of BP consistently and continuously reduces cardiovascular risk. This is supported by the Royal College of Physicians (RCP) guidelines’ aim for BP <140/90 mm Hg, with an ideal target BP <130/80 mm Hg for secondary prevention.^29^ The effect of intervention on BP reduction using 24-hour BP measurement at the beginning and end of the study will be examined. This will be recorded using a SpaceLabs 90 207 monitor programmed to measure BP at 20 min intervals during the daytime (07:00–22:00 hours) and hourly overnight (22:00–07:00 hours).

### Plasma lipids

The link between total cholesterol and dementia is controversial, but from a cardiovascular risk factor point of view lowering cholesterol by using statins improves stroke secondary prevention. Although poststroke dementia can be of VaD type, Alzheimer’s dementia or mixed pathology, lowering cholesterol should contribute to a reduced risk of poststroke dementia where the mechanism is one of VaD. Therefore, the cholesterol aim has been chosen as one of the ‘treat-to-target’ interventions. The total cholesterol aim will be <4.0 mmol/L, which is in line with the RCP guidelines which were current at the trial inception.^29^

### Atrial fibrillation

It has been shown that people with stroke and AF are more likely to be subsequently diagnosed with dementia.^3 5^ An intervention rate aim of 60–80 beats per minute has been chosen for patients with AF; those on warfarin will aim for an international normalised ratio (INR) between 2.5 and 3 to maintain levels in the therapeutic window. There are no specific drug monitoring targets for other anticoagulants.

Ten minutes of continuous beat-to-beat BP measurement will be carried out at baseline and the final follow-up using a Finometer device. These data can be used to assess heart rate variability as well as BP variability.

### Blood glucose

Diabetes mellitus is associated with both microvascular and macrovascular disease, and hence carotid artery disease which is a preventable risk factor for stroke. It has been well documented that poor glucose control (assessed using haemoglobin A1c (HbA1c)) predicts stroke risk.^30^ Therefore, good diabetes control may prevent further cardiovascular risk and be associated with added benefit to future cognitive status. The aim is for HbA1c of 48–53 mmol/mol (or 6.5%–7%).

The patient’s GP will be informed by letter of the results that have been recorded during the research study. All patients will receive standard lifestyle advice relating to diet and weight, smoking and alcohol consumption.

### Ordering of assessments

The following assessments will be carried out on all participants at baseline (table 1):

- Eligibility assessment and informed consent;
- MoCA;
- Demographics, including age, gender, body mass index, smoking status, alcohol consumption and exercise habits;
- Assessment of past medical history, including VRF;
- Record concomitant medications.

**Table 1:** Summary of study procedures

Procedures for all participants	Visits					
	Screening	Baseline	3 months	6 months	9 months	12 months
Eligibility assessment	✓	✓	–	–	–	✓
Informed consent	✓	✓	–	–	–	–
Montreal Cognitive Assessment	✓	–	–	–	–	✓
Medical history	✓	–	–	–	–	–
Demographics	–	✓	–	–	–	–
Concomitant medications	–	✓	–	–	–	✓
Physical examination including VRFs	–	✓	–	–	–	✓
Blood sample for cholesterol±INR and blood glucose/HbA1c	–	✓	–	–	–	✓
Adverse events	–	–	–	–	–	✓
Additional procedures for participants in group C						
24-hour BP measurement	–	✓	–	–	–	✓
Beat-to-beat BP measurement	–	✓	–	–	–	✓
Pulse wave velocity measurement	–	✓	–	–	–	✓
Quality of life and functional assessment*	–	✓	–	–	–	✓
Resource use questionnaires	–	✓	–	–	–	✓
Additional procedures for participants in group I						
Eligibility assessment	–	–	✓	✓	✓	–
Assessment of VRFs	–	–	✓	✓	✓	v
Blood sample for cholesterol±INR and blood glucose/HbA1c	–	–	✓	✓	✓	–
Concomitant medications and adherence	–	–	✓	✓	✓	–
Quality of life and functional assessment*	–	✓	–	–	–	✓
Resource use questionnaires	–	✓	–	–	–	✓
24-hour BP measurement	–	✓	–	–	–	✓
Beat-to-beat BP measurement	–	✓	–	–	–	✓
Pulse wave velocity measurement	–	✓	–	–	–	✓
Adverse events	–	–	✓	✓	✓	–

*Includes EQ5D, Dementia Quality of Life measure, Geriatric Depression Scale, Bristol Activities of Daily Living, Morisky Medication Score.

BP, blood pressure; HbA1c, haemoglobin A1c; VRF, vascular risk factor.

In addition, participants in groups C and I will complete at baseline:

- EQ5D (a generic health-related quality of life questionnaire), DEMQOL (Dementia Quality of Life measure), GDS (Geriatric Depression Scale questionnaire), Bristol Activities of Daily Living questionnaire and Morisky Medication Adherence Score;
- Resource use questionnaires;
- BP variability measures including 10 min of continuous beat-to-beat BP recording and 24-hour BP monitoring;
- Pulse wave velocity (PWV) measurements (which reflect arterial stiffness).

Participants in group I will be seen at 3, 6 and 9 months at which time they will have assessment of their VRF and data collection for adverse events, including recurrent stroke/TIA.

All participants will be followed up at 12 months at which time they will have:

- MoCA;
- Assessment of VRF;
- Recording of concomitant medications;
- Data collection for adverse events, including recurrent stroke/TIA.

In addition, participants in group C and I will complete at 12 months:

- EQ5D, DEMQOL, GDS, Bristol Activities of Daily Living questionnaire and Morisky Medication Adherence Score;
- Resource use questionnaires;
- BP variability measures;
- PWV measurements.

### Outcome measurements

#### Primary outcome measure

Recruitment and retention rates at 12 months from the screening and study management logs.

#### Secondary outcome measures

- Difference in mean change in MoCA score between groups C and I at 12 months;
- Proportions of participants in each group whose vascular risk factors are controlled at each time point;
- Frequency of adverse events in each group;
- Indicative incremental cost per MoCA point and quality-adjusted life-year (QALY) gained by the intervention;
- Mean change in MoCA score in group O related to number of VRF and proportion of participants whose VRF are controlled at baseline and outcome.

### Sample size calculation

As this is a feasibility study a formal sample size calculation has not been performed. The duration and sample size of the study are based on the estimated prevalence rate of cognitive impairment at diagnosis (around 30%),^4^ incidence of dementia after the event (~30% in 3 months),^3^ estimated screening and recruitment rates. The aim is to include a minimum of 100 patients in the feasibility study (50 per group) and another 100 patients in the observational study.

### Data analysis plan

#### Primary objective

- Proportion of participants with MoCA score 20–25 who consent to join the trial.
- Adherence to follow-up, including rates of withdrawal and loss to follow-up.
- Number of risk factors that need to be targeted in these patients administering the client service receipt inventory (CSRI) and quality of life questionnaires. Originally designed for costing psychiatric interventions, the CSRI^31^ has been used as the core resource use measurement tool for a wide variety of interventions. It requires adapting to fit each study question, therefore the feasibility study will allow testing and revising of the questionnaire in preparation for the full study.

#### Secondary objectives

- Rates of control of VRF at baseline and outcome in each group.
- Proportion of participants in group I achieving VRF targets at 3, 6 and 9 months for each risk factor.
- Difference in mean change in MoCA score between groups C and I. The between-group comparison will be based on a general linear model with group as a fixed effect and including any prognostic variables at baseline for which there is a between-group disparity. A 95% CI for the difference in means will be constructed to give an idea of the likely magnitude of benefit from the intervention.
- Indicative incremental cost per point gained in MoCA, DEMQOL and per QALY gained between groups C and I. Data will be analysed in terms of costs and effects for the two groups. We will analyse key drivers of costs and examine the potential of this intervention to be cost-effective.
- Change in MoCA score between baseline and 12 months in group O participants, and in groups O and C combined.
- Difference in adverse event rates between groups.
- Difference in mean BP between groups C and I.
- Difference in BP (systolic and diastolic) variability between groups C and I.
- Difference in PWV between groups C and I.

### Ethics and dissemination

Study oversight will be conducted through regular meetings of a Trial Steering Committee and a separate Safety Committee, both of which will include independent representatives. If it is felt that the risk to participants is significant or unacceptable the Safety Committee can recommend to early termination of the trial.

Data will be collected and handled in line with sponsor and Norwich CRTU procedures and NHS Trust policies. Electronic data will be anonymised and all data will be kept under secure conditions. Professor Potter will act as data custodian.

Dissemination of the study results is planned via publication in peer-reviewed medical journals and presentation at relevant scientific conferences. Any reporting will adhere to the Consolidated Standards of Reporting Trials statement extension for pilot and feasibility trials. We do not intend to employ professional writers.

## Supplementary Material

Reviewer comments

Author's manuscript
