# Supplementary material for: Protocol for a feasibility randomised controlled trial of Screening and Enhanced Risk management for Vascular Event-related Decline in Memory (SERVED Memory)
Source: BMJ Open. 2017 Nov 28;7(11):e017416. doi: 10.1136/bmjopen-2017-017416 (PMC5719289; doi:10.1136/bmjopen-2017-017416)
Supplement: Author's manuscript [file bmjopen-2017-017416.draft_revisions.pdf]

BMJ Open is committed to open peer review. As part of this commitment we make the peer review history of every article we publish publicly available.

When an article is published we post the peer reviewers' comments and the authors' responses online. We also post the versions of the paper that were used during peer review. These are the versions that the peer review comments apply to.

The versions of the paper that follow are the versions that were submitted during the peer review process. They are not the versions of record or the final published versions. They should not be cited or distributed as the published version of this manuscript.

BMJ Open is an open access journal and the full, final, typeset and author-corrected version of record of the manuscript is available on our site with no access controls, subscription charges or pay-per-view fees (http://bmjopen.bmj.com).

If you have any questions on BMJ Open's open peer review process please email editorial.bmjopen@bmj.com

BMJ Open

Feasibility Randomised Controlled Trial of Screening & Enhanced Risk Management for Vascular Event related Decline in Memory (SERVED Memory)

Journal:	*BMJ Open*
Manuscript ID	bmjopen-2017-017416
Article Type:	Protocol
Date Submitted by the Author:	22-Apr-2017
Complete List of Authors:	Myint, Phyo; University of East Anglia, School of Medicine, Medical Sciences & Nutrition<br>Loke, Yoon; University of East Anglia, Norwich Medical School<br>Davison, William; University of East Anglia Norwich Medical School<br>Mattishent, Katharina; University of East Anglia, Norwich Medical School<br>Fox, Chris; Norwich Medical School, Department of Psychological Sciences<br>Fleetcroft, Robert; University of East Anglia<br>Turner, David; University of East Anglia, Public Health and Primary Care<br>Shepstone, Lee; University of East Anglia<br>Potter, John; University of East Anglia, Norwich Medical School; Norfolk and Norwich University Hospital, Stroke Research Group
**Primary Subject Heading**:	Cardiovascular medicine
Secondary Subject Heading:	Neurology, Geriatric medicine
Keywords:	Stroke < NEUROLOGY, Dementia < NEUROLOGY, VASCULAR MEDICINE, STROKE MEDICINE

SCHOLARONE™
Manuscripts

For peer review only

Protocol (3/5/16 v3.3)

Feasibility Randomised Controlled Trial of Screening & Enhanced Risk Management for Vascular Event related Decline in Memory (SERVED Memory)

Correspondence to:

Professor Phyo Kyaw Myint

Room 4.013, Polwarth Building

Foresterhill, University of Aberdeen

Aberdeen, AB25 2ZD

UK

Tel: + 44 (0) 1224 437841

Fax: +44 (0) 1224 437911

Mail to: phyo.myint@abdn.ac.uk

Sponsor:

Laura Harper

Research Study and Recruitment Facilitator

Norfolk and Norwich University Hospitals NHS Foundation Trust

Colney Lane

Norwich, NR4 7UY

Tel: +44 (0) 1603 287408

Fax: +44 (0) 1603 288314

Mail to: laura.harper@nnuh.nhs.uk

Keywords: cognitive impairment, dementia, vascular dementia, stroke, TIA

Word count (excl title page, refs, tables, figures): 3994 words

Authors:

Professor Phyo Kyaw Myint, Professor of Medicine of Old Age, University of Aberdeen, Aberdeen, UK

Professor Yoon K Loke, Professor of Medicine & Pharmacology, University of East Anglia, Norwich, UK

Dr William Davison, Clinical Research Fellow in Stroke Medicine, University of East Anglia, Norwich, UK

For peer review only

Dr Katharina Mattishent, Clinical Research Fellow in Older Peoples' Medicine, University of East Anglia, Norwich, UK

Professor George Christopher Fox, Professor of Psychiatry, University of East Anglia, Norwich, UK

Dr Robert Fleetcroft, General Practitioner and Honorary Senior Fellow, Norwich Medical School, Norwich, UK

Mr David Turner, Senior Researcher in Health Economics, Norwich Medical School, Norwich, UK

Professor Lee Shepstone, Professor of Medical Statistics, University of East Anglia, Norwich, UK

Professor John F Potter, Professor of Ageing & Stroke Medicine, University of East Anglia, Norwich, UK

Author Contributions:

P Myint, J Potter, Y Loke, G Fox, R Fleetcroft, D Turner and L Shepstone were involved in the original conception and design of the study. The original protocol was written by P Myint, Y Loke and K Mattishent, and reviewed by the other authors listed above.

W Davison is part of the research team involved in recruitment and data acquisition and was responsible for revising the protocol for submission to BMJ Open, with input from all other contributing authors.

The final manuscript has been reviewed by all authors and approved for submission/publication.

For peer review only

ABSTRACT

Introduction: Stroke is a leading cause of death and disability. The development of dementia after stroke is common. Vascular risk factors (VRF) which contribute to stroke risk also contribute to cognitive decline, especially in vascular dementia (VaD). There is no established treatment for VaD, therefore strategies for prevention could have major health resource implications. This study was designed to assess whether patients with early cognitive decline after stroke/transient ischaemic attack (TIA) can be easily identified and whether target driven VRF management can prevent progression to dementia.

The study's primary objective is to establish the feasibility of recruiting patients with mild cognitive impairment (MCI) to a randomised controlled trial of enhanced VRF management. Secondary objectives include (a) to determine the potential clinical benefit of the intervention; (b) to estimate the sample size for a future definitive multi-centre randomised controlled trial; (c) to inform a future economic evaluation of the intervention; (d) to explore the link between VRF control and the incidence of cognitive impairment on longitudinal follow-up in a UK population after stroke/TIA with current routine management.

Methods: 100 patients with MCI post-stroke/TIA will be recruited from stroke services at the Norfolk and Norwich University Hospital (NNUH). After collection of baseline data they will be randomised to intervention (3 monthly follow-up with enhanced management) or control (treatment as usual by the General Practitioner (GP)). At 12 months outcomes (repeat cognitive testing, VRF assessment) will be assessed. A further 100 patients with normal cognition will be recruited to a parallel observational group from the same site. At 12 months they will have repeat cognitive testing.

Ethics and dissemination: Ethical approval has been granted in England. Dissemination is planned via publication in peer-reviewed medical journals and presentation at relevant conferences.

Registration details: International standard randomised controlled trial number (ISRCTN) 42688361.

For peer review only

Strengths and limitations of this study:

- The protocol utilises a validated cognitive screening test which is sensitive and specific for the detection of mild cognitive impairment as well as dementia.
- Data will be collected on a range of VRF.
- A limitation of the study is that neither research staff nor participants are blinded to the intervention.

INTRODUCTION

Background

Stroke is one of the leading causes of death and disability. Current demographic trends suggest that the total numbers of people with a stroke will rise due to the ageing population (Myint et al, 2008); with significant concerns regarding rising incidence of VaD for which there is at present no established treatment. Dementia after stroke poses a significant problem considering that up to 30% of people with stroke may potentially develop dementia as early as 3-months post stroke (Kwok et al, 2011). Furthermore, a significant proportion of these patients have already had MCI (Guyomard et al, 2011).

The World Alzheimer Report emphasised the benefit of early diagnosis with future savings from delayed institutionalisation, and care costs across the disease course (World Alzheimer report, 2010). Similarly the UK Government has identified the timely diagnosis of dementia in primary care as a priority (National Audit Report, 2005). An effective strategy in preventing VaD could have major resource implications - with at least 1 in 5 dementia cases having a VaD element and dementia costing the UK economy £23 billion per year (Dementia, 2010). Most importantly, "*how best to improve cognition after stroke"* was reported to be the highest priority research topic in a survey of patients with stroke (Pollock et al., 2012).

Vascular risk factors (e.g. high blood pressure and diabetes) can contribute to the cognitive decline in VaD as well as in mixed dementia. Research suggests they play a key role in the development of cognitive decline. Our recent work shows that this risk appears to be greater in people with higher numbers of VRF (Guyomard et al, 2011). The most recent literature also suggests that the conversion of MCI to dementia is more likely in patients who have cardiovascular risk factors (Ettorre et al, 2012). From published literature (Allan et al., 2011) and our preliminary work (Guyomard et al., 2011; Kwok et al., 2011) it is known that dementia is common after stroke and TIA. However, to date

For peer review only

little specific attempt has been made in developing an intervention to reduce this risk after such an event.

Rationale for the Study

Research studies report a variable incidence of dementia post-stroke with the highest rates being around 30% (Leys et al., 2005). Reporting differences are mainly attributed to variation in cohort ages and the diagnostic criteria applied. However, there does not seem to be any similar data based on the current UK population. Therefore, it is important to conduct a feasibility study with a parallel observational design in the UK NHS setting. The scientific hypothesis underpinning this feasibility randomised controlled trial, with an observational study embedded within it, is that detection of early cognitive decline in stroke and TIA is feasible at the time of diagnosis in secondary care and that enhanced (target driven) multiple risk factor control is clinically effective, cost-effective and safe. Routine cognitive testing using validated measures which are simple and quick, such as the Montreal Cognitive Assessment (MoCA) (Nasreddine et al.,2005), which are shown to be sensitive and specific in detecting vascular related cognition will identify those who are at risk of developing decline. The rate of decline in cognitive function is most likely to be influenced by the type of risk factors and the level of risk factor control (e.g. adequate level of anticoagulation for AF/control of BP). Furthermore, there is a dearth of information specifically relevant to patients with cognitive deficit but not necessarily being diagnosed as having MCI. Further observational epidemiology may provide better insight regarding cognition after stroke/TIA in this patient population.

Study objectives

The primary objective of the study is to determine the feasibility of randomising patients who have MCI into routine risk factor management or enhanced risk factor management by their GP, with individualised targets for controllable risk factors.

The secondary objectives are:

(a) to determine the potential clinical benefit of enhanced control of VRF in preventing progression of cognitive decline and the development of dementia;

(b) to assess indicative cost-effectiveness of this intervention;

(c) to estimate the sample size for a future definitive multi-centre randomised controlled trial;

(d) to identify any adverse events due to the intervention;

(e) to explore the incidence of cognitive impairment on longitudinal follow-up in a UK population after stroke/TIA with current routine risk factor management.

For peer review only

METHODS AND ANALYSIS

Study overview

This study is a single-centre, open-label parallel group study to determine the feasibility of conducting a randomised controlled trial in an NHS setting on patients following stroke or TIA who have MCI. The aim is to target risk factors more intensively through enhanced monitoring and control of VRF compared to usual care. We wish to estimate the potential clinical impact and cost-effectiveness of this intervention, and the sample size for a future multicentre definitive study in an NHS setting.

There is a parallel observational study arm for patients with no evidence of cognitive decline who will have their VRF and cognitive function assessed at follow-up. The objective of the parallel observational study is to better understand the link between VRF, their control and the development of cognitive decline after a cerebrovascular event. Combining the control arm of the feasibility trial and observational cohort will provide further information on these links, including a realistic estimate of the magnitude of effect of enhanced risk factor management in planning a future trial. A summary of the study design is provided in **Figure 1.** Recruitment commenced in November 2015 and will complete in July 2017, with final follow-up data collection in July 2018. The study has been registered: International standard randomised controlled trial number (ISRCTN) 42688361.

Trial Participants

All adult patients with confirmed stroke (first/recurrent) or TIA, identified within 8 weeks of diagnosis will be considered for the trial.

Inclusion Criteria

- Participant is willing and able to give informed consent for participation;
- Male or female, aged 18 years or above;
- Diagnosed clinically and radiologically with stroke (infarct or haemorrhage) or TIA.

Exclusion Criteria

The participant may not enter the trial if ANY of the following apply:

- Established Dementia;
- Life expectancy <1 year;
- Co-morbidities that adversely affect their ability to accurately complete the MoCA;
- Patients who do not wish to know about their cognition.

For peer review only

Trial procedures including recruitment, randomisation, baseline and follow-up assessments are summarised in **Figures 2 and 3**.

Identification of participants

Patients diagnosed with Stroke/TIA within 8 weeks of onset who are considered by the healthcare team to be eligible for the study will be invited to participate. They will be given a detailed Patient Information Leaflet and Consent Form for consideration. The study team will contact the patient after 24 hours to find out whether or not they are willing to participate. Those who agree and provide written informed consent will undergo a simple and validated cognitive screening test (MoCA), unless this has already been carried out by the clinical team as part of their routine care, whereupon that score can be used for study purposes. If a MoCA has been administered by the TIA clinic team the patient will be given a PIS in clinic and be followed up by phone to discuss entry into the study. Patients who have previously attended TIA clinic or a stroke ward as an in-patient may be screened retrospectively from the Capture TIA/Stroke hospital database and followed up by phone. Those who have a MoCA score ≥26 since their stroke or TIA and who verbally consent to be contacted about the study are sent a study summary sheet, a study invitation letter and consent form. If they wish to participate the completed consent form will be returned and countersigned by a delegated member of the study team. Those who do not have a MoCA score following their stroke or TIA are invited for an appointment to give consent to take part in the study and carry out the MoCA. The patient will be enrolled into the appropriate arm of the study depending on the MoCA score.

Assessing capacity and obtaining informed consent

The participant must personally sign and date the latest approved version of the Informed Consent Form. It will then be countersigned by a delegated member of the research team before any trial specific procedures are performed. Written and verbal versions of the patient information sheet will be presented to the participants detailing the trial rationale; participant involvement and responsibilities; the implications and constraints of the protocol; safeguards; and processing of blood tests. It will be clearly stated that the participant is free to withdraw from the trial at any time for any reason without prejudice to future care, and with no obligation to give the reason for withdrawal. The participant will be allowed at least 24 hours to consider the information and have the opportunity to ask questions before deciding whether they will participate in the trial. The member of the research team who takes consent will be delegated to do so, be familiar with the study and be suitably qualified to obtain consent for research purposes. A copy of the signed

For peer review only

informed consent will be given to the participant, and another will be stored at NNUH Clinical Research Trials Unit (CRTU). The original signed form will be retained in the patient's medical records.

Interventions to be measured

The proposed intervention is based on the current evidence of the impact of risk factor control on cognition as well as reduction in further cardiovascular events. All chosen risk factors in this study have been shown to be linked with dementia risk. The aim is to provide further evidence that enhanced monitoring and treatment of these risk factors would halt or attenuate cognitive decline, or even potentially improve cognition in a specific patient population who are at high risk of developing dementia. A recent randomised controlled trial in Germany also demonstrated a significant reduction in the need for long-term care in older adults following an intervention involving systematic identification and evidence-based treatment of cardiovascular risk factors (Bickel et al, 2012).

The Vascular Risk Factors that will be closely monitored by the study team will be:

Blood pressure (BP)

Existing evidence demonstrates that lowering of blood pressure consistently and continuously reduces cardiovascular risk. This is supported by the current Royal College of Physicians (RCP) guidelines' aim for BP 140/90 mmHg, with an ideal target BP <130/80 mmHg for secondary prevention. The effect of intervention on BP reduction using 24 hour BP measurement at the beginning and end of the study will be examined. This will be recorded using a SpaceLabs 90207 monitor programmed to measure BP at 20 minute intervals during the daytime (0700-2200) and hourly overnight (2200-0700).

Plasma Lipids

The link between total cholesterol and dementia is controversial, but from a cardiovascular risk factor point of view lowering cholesterol by using statins improves stroke secondary prevention. This is particularly important because VaD progresses in a step-wise manner (i.e. multi-infarct dementia). Therefore, the cholesterol aim has been chosen as one of the "treat to target" interventions. The total cholesterol aim will be <4.0 mmol/L which is in line with the RCP guidelines which were current at the trial inception.

Atrial fibrillation (AF)

It has been shown that people with stroke and AF are more likely to be subsequently diagnosed with dementia. An intervention rate aim of 60-80 beats per minute has been chosen for patients with AF;

For peer review only - http://bmjopen.bmj.com/site/about/guidelines.xhtml

those on warfarin will aim for an INR between 2.5 and 3 to maintain levels in the therapeutic window. There are no specific drug monitoring targets for other anticoagulants.

10 minutes of continuous beat-to-beat BP measurement will be carried out at baseline and the final follow-up using a Finometer device. This data can be used to assess heart rate variability as well as blood pressure variability.

Blood glucose

Diabetes mellitus is associated with both micro and macrovascular disease, and hence carotid artery disease which is a preventable risk factor for stroke. It has been well documented that poor glucose control (assessed using HbA1C) predicts stroke risk (Myint et al, 2007). Therefore good diabetes control may prevent further cardiovascular risk and be associated with added benefit to future cognitive status. The aim is for HbA1C of 48-53 mmol/mol (or 6.5-7%).

The patient's GP will be kept informed, by letter, of the results that have been recorded during the research study. All patients will receive standard lifestyle advice relating to diet and weight, smoking, and alcohol consumption.

Allocation into Study Arms

This will take place after recording the participant's MoCA score, which is interpreted in the following manner:

- Score ≥26 indicates normal cognition whilst a score ≤17 suggests possibility of dementia.
- Score between 20-25 suggests MCI, which is an Intermediate stage between the expected cognitive decline of normal ageing and the more serious decline of dementia.

The allocation of participants based on their MOCA score is as follows:

Greater degree of cognitive decline (MoCA <20)

In view of the greater extent of cognitive impairment, continued participation in the study is not suitable for these participants. They will be referred to specialist services where relevant, and their GP will be informed.

Normal cognition (MoCA score ≥26)

Patients with normal cognition (MoCA score ≥26) will be informed of their normal cognition status and will continue to receive usual care by their clinicians. They will be asked to confirm their willingness to continue to participate in the observational study **(GROUP O)** and will be followed-up at 12 months.

Intermediate stage (MoCA score 20-25)

For peer review only

Patients with MoCA score of 20-25 will be informed of their cognitive status and asked to confirm their willingness to continue to participate in the feasibility trial. They will be randomised into one of two groups; control arm **(GROUP C)** or intervention arm **(GROUP I)**. Randomisation will be based on computer generated blocked randomisation managed by the Norwich CRTU.

GROUP C: The patients will receive usual care by their clinicians and will be followed-up at 12 months.

GROUP I: Patients in this arm will undergo enhanced risk factor management through assessment by the study team at 3, 6, and 9 months. Specific aims will be set for each modifiable risk factor and the GP will be informed about these targets and the results of each visit.

Ordering of assessments

The following assessments will be carried out on all participants at baseline:

- Eligibility assessment and informed consent;
- MoCA;
- Assessment of past medical history, including VRF;
- Record concomitant medications.

In addition, participants in group C and I will complete at baseline:

- EQ5D (a generic health-related quality of life questionnaire), DEMQOL (a dementia-specific QoL questionnaire), GDS (Geriatric Depression Scale questionnaire), Bristol Activities of Daily Living questionnaire and Morisky Medication Adherence Score;
- Resource use questionnaires;
- BP variability measures including 10 minutes of continuous beat-to-beat blood pressure recording and 24 hour blood pressure monitoring;
- Pulse wave velocity (PWV) measurements (which reflect arterial stiffness).

Participants in group I will be seen at 3, 6, and 9 months at which time they will have assessment of their vascular risk factors and data collection for adverse events.

All participants will be followed-up at 12 months at which time they will have:

- MoCA;
- Assessment of vascular risk factors;
- Recording of concomitant medications;

For peer review only

In addition, participants in group C and I will complete at 12 months:

- EQ5D, DEMQOL, GDS, Bristol Activities of Daily Living questionnaire and Morisky Medication Adherence Score;
- Resource use questionnaires;
- Data collection for adverse events;
- BP variability measures;
- PWV measurements.

A summary of these assessments is presented in **Tables 1 and 2**.

Outcome measurements (what, when, how)

1. Primary outcome measure:

Recruitment and retention rates at 12 months from the screening and study management logs.

2. Secondary outcome measures:

(a) difference in mean change in MoCA score between groups C and I at 12 months;

(b) proportions of participants in each group whose vascular risk factors are controlled at each time point;

(c) frequency of adverse events in each group;

(d) indicative incremental cost per MoCA point and QALY gained by the intervention;

(e) mean change in MoCA score in group O related to number of VRF and proportion of participants whose VRF are controlled at baseline and outcome.

Coding and recording assessments

All trial data will be entered in paper Case Record Forms. These will be stored in locked offices, which require passcode access, within the NNUH CRTU. Anonymised data will be transcribed to a secure database by the researchers or a suitably qualified member of the research team. This database will be stored on password protected computers at the NNUH CRTU. The name and any other identifying detail will not be included in any trial data electronic file.

Sample size calculation

As this is a feasibility study a formal sample size calculation is not required. The duration and sample size of the study are based on the estimated prevalence rate of cognitive impairment at diagnosis

For peer review only

(around 30% (Guyomard et al, 2011)), incidence of dementia after the event (~30% in 3 months (Kwok et al, 2011)), estimated screening and recruitment rates. The aim is to include a minimum of 100 patients in the feasibility study (50 per group) and another 100 patients in the observational study.

Data analysis plan

Primary objective:

- Proportion of participants with MoCA score 20-25 who consent to join the trial;
- Adherence to follow-up, including rates of withdrawal and loss to follow-up;
- Number of risk factors that need to be targeted in these patients administering the client service receipt inventory (CSRI) and Quality of Life questionnaires. Originally designed for costing psychiatric interventions, the CSRI (Chisholm et al, 2000; Beecham & Knapp, 2001) has been used as the core resource use measurement tool for a wide variety of interventions. It requires adapting to fit each study question therefore the feasibility study will allow testing and revising of the questionnaire in preparation for the full study.

Secondary objectives:

- Rates of control of VRF at baseline and outcome in each group;
- Proportion of participants in group I achieving VRF targets at 3,6 and 9 months for each risk factor;
- Difference in mean change in MoCA score between groups C and I. The between group comparison will be based on a general linear model with group as a fixed effect and including any prognostic variables at baseline for which there is a between group disparity. A 95% confidence interval for the difference in means will be constructed to give an idea of the likely magnitude of benefit from the intervention;
- Indicative incremental cost per point gained in MoCA, DEMQOL and per QALY gained between Group C and I. Data will be analysed in terms of costs and effects for the two groups. We will analyse key drivers of costs and examine the potential of this intervention to be cost-effective.
- Change in MoCA score between baseline and 12 months in GROUP O participants, and in groups O and C combined;
- Difference in adverse events rates between groups;
- Difference in mean BP between Groups C and I;
- Difference in BP (systolic and diastolic) variability between Groups C and I;
- Difference in PWV between Groups C and I.

For peer review only

ETHICS

This study was granted ethical approval in England (East of England Cambridge East Research Ethics Committee, REC 15/EE/0061).

Study oversight

Study oversight is through the Trial Steering Committee, which will meet every four months. The Trial Steering Committee comprises two independent lay representatives, two independent experts (one of whom is the Chair of the Committee), the PI, the study statistician, and representatives from the Norwich CRTU. There will also be a Safety Committee (in place of a data monitoring committee), comprising two independent clinicians, who will meet every six months to evaluate any unexpected trends or unexpected risk to participants. If it is felt that the risk to participants is significant or unacceptable the Safety Committee can recommend to early termination of the trial.

Data Protection

Data will be collected and handled in line with sponsor and Norwich CRTU Standard Operating Procedures and NHS Trust policies. All electronic data will be link-anonymised.

DISCUSSION

This study was designed to assess the feasibility of recruiting patients with MCI post-stroke/TIA to a randomised controlled trial of enhanced VRF management. The main challenge of the study so far has been recruitment. Initially this was slow due to the primary research nurse having an additional active study. Following the conclusion of the parallel study it has been possible to focus on SERVED Memory exclusively and, with an additional research fellow joining the team, recruitment rates have improved. However, whilst recruitment to group O has not been problematic, we have found that patients with MoCA score 20-25 are less likely to consent to participate. They are often older, have a greater degree of frailty, and are more greatly affected as a result of their stroke. Consequently the perceived burden of participation is likely to be greater. As this is a feasibility study we have not made any alterations to the intervention at this stage as we want to quantify the recruitment and retention rates, including reasons for non-recruitment and withdrawal. This data will be important for designing the future definitive study where it may be necessary to adjust the follow-up, for example by increasing the interval between visits or offering follow-up in the patient’s home.

For peer review only

CONCLUSION

SERVED Memory aims to demonstrate the feasibility of identifying patients with MCI post-stroke/TIA and recruiting them to a trial of enhanced VRF management. We also aim to show that enhanced VRF management can prevent further cognitive deterioration, and potentially even improve cognitive function in stroke survivors, and that this intervention is acceptable. A health economic component will explore the capacity of the intervention to be cost-effective. We hope that this will inform future study with the ultimate goal of developing realistic strategies for preventing VaD and other types of dementia, which in turn could have a major impact on patient care and healthcare resources.

Conflict of interest

None.

Acknowledgement

We gratefully acknowledge the funding organisation for this feasibility trial, NIHR Research for Patient Benefit Programme (DRF-2013-06-115).

For peer review only

References

Allan LM, Rowan EN, Firbank MJ, Thomas AJ, Parry SW, Polvikoski TM, O'Brien JT, Kalaria RN. Long term incidence of dementia, predictors of mortality and pathological diagnosis in older stroke survivors. Brain. 2011;134:3716-27.

Beecham J, Knapp M. Costing psychiatric interventions. In Measuring Mental Health Needs (2nd edn) (ed. G. Thornicroft) 2001; pp. 200-224. Gaskell.

Bickel H, Ander KH, Brönner M, Etgen T, Gnahn H, Gotzler O, Poppert H, Pürner K, Sander D, Förstl H. Reduction of Long-Term Care Dependence After an 8-Year Primary Care Prevention Program for Stroke and Dementia: The INVADE Trial. J Am Heart Assoc. 2012 Aug;1(4).

Bryan S, Hardyman W, Bentham P, Buckley A, Laight A. Proxy completion of EQ-5D in patients with dementia. Qual Life Res. 2005;14:107-18.

Dementia 2010- the prevalence, economic cost, research funding of dementia compared with other major diseases. http://www.dementia2010.org/

Dolan P. Modeling valuations for EuroQol health states. Med Care. 1997;35:1095-108.

Ettorre E, Cerra E, Marigliano B, Vigliotta M, Vulcano A, Fossati C, De Benedetto G, Servello A, Andreozzi P, Marigliano V. Role of cardiovascular risk factors (CRF) in the patients with mild cognitive impairment (MCI). Arch Gerontol Geriatr. 2012 Mar-Apr;54(2):330-2.

Greenberg SM, Eng JA, Ning M, Smith EE, Rosand J. Hemorrhage burden predicts recurrent intracerebral hemorrhage after lobar hemorrhage. Stroke. 2004;35:1415–20.

Gorelick PB, Scuteri A, Black SE, Decarli C, Greenberg SM, Iadecola C, Launer LJ, Laurent S, Lopez OL, Nyenhuis D, Petersen RC, Schneider JA, Tzourio C, Arnett DK, Bennett DA, Chui HC, Higashida RT, Lindquist R, Nilsson PM, Roman GC, Sellke FW, Seshadri S; on behalf of the American Heart Association Stroke Council, Council on Epidemiology and Prevention, Council on Cardiovascular Nursing, Council on Cardiovascular Radiology and Intervention, and Council on Cardiovascular Surgery and Anesthesia. Vascular Contributions to Cognitive Impairment and Dementia: A Statement for Healthcare Professionals From the American Heart Association/American Stroke Association. Stroke. 2011;42:2672-713.

Guyomard V, Metcalf AK, Naguib MF, Fulcher RA, Potter JF, Myint PK. Transient ischaemic attack, vascular risk factors and cognitive impairment: a case-controlled study. Age Ageing. 2011;40:641-4.

Kwok CS, Loke YK, Hale R, Potter JF, Myint PK. Atrial fibrillation and incidence of dementia: a systematic review and meta-analysis. Neurology. 2011;76:914-22.

Kwok CS, Skinner J, Metcalf AK, Potter JF, Myint PK. Prior antiplatelet and anticoagulant therapy and mortality in stroke. Heart. 2012;98:712-7.

For peer review only

Leys D, Henon H, Mackowiak-Cordoliani MA, Pasquier F. Poststroke dementia. Lancet Neurol. 2005;4:752-59.

Myint PK, Sinha S, Wareham NJ, Bingham SA, Luben RN, Welch AA, Khaw KT. Glycated hemoglobin and risk of stroke in people without known diabetes in the European Prospective Investigation into Cancer (EPIC)-Norfolk prospective population study: a threshold relationship? Stroke. 2007 Feb;38(2):271-5.

Myint PK, Sinha S, Luben RN, Bingham SA, Wareham NJ, Khaw KT. Risk factors for firstever stroke in the EPIC-Norfolk prospective population-based study. Eur J Cardiovasc Prev Rehabil. 2008; 15:663-9.

Nasreddine ZS, Phillips NA, Bedirian V. The Montreal Cognitive Assessment, MoCA: a brief screening tool for mild cognitive impairment. J Am Geriatr Soc. 2005;53:695-9.

National Audit Report. Department of Health - Reducing Brain Damage: Faster access to better stroke care. 2005.

Pendlebury ST, Mariz J, Bull L, Mehta Z, Rothwell PM. MoCA, ACE-R, and MMSE Versus the National Institute of Neurological Disorders and Stroke-Canadian Stroke Network Vascular Cognitive Impairment Harmonization Standards Neuropsychological Battery After TIA and Stroke. Stroke. 2012 ;43:464-9.

Pollock A, St George B, Fenton M, Firkins L. Top ten research priorities relating to life after stroke. Lancet Neurol. 2012;11:209.

Ritchie J & Spencer L. Qualitative data analysis for applied policy research. In Analysing Qualitative Data,pp. 173–194 (eds. A Bryman and RG Burgess). London: Routledge, 1994.

World Alzheimer's Report 2010 http://www.alz.co.uk/research/world-report

For peer review only

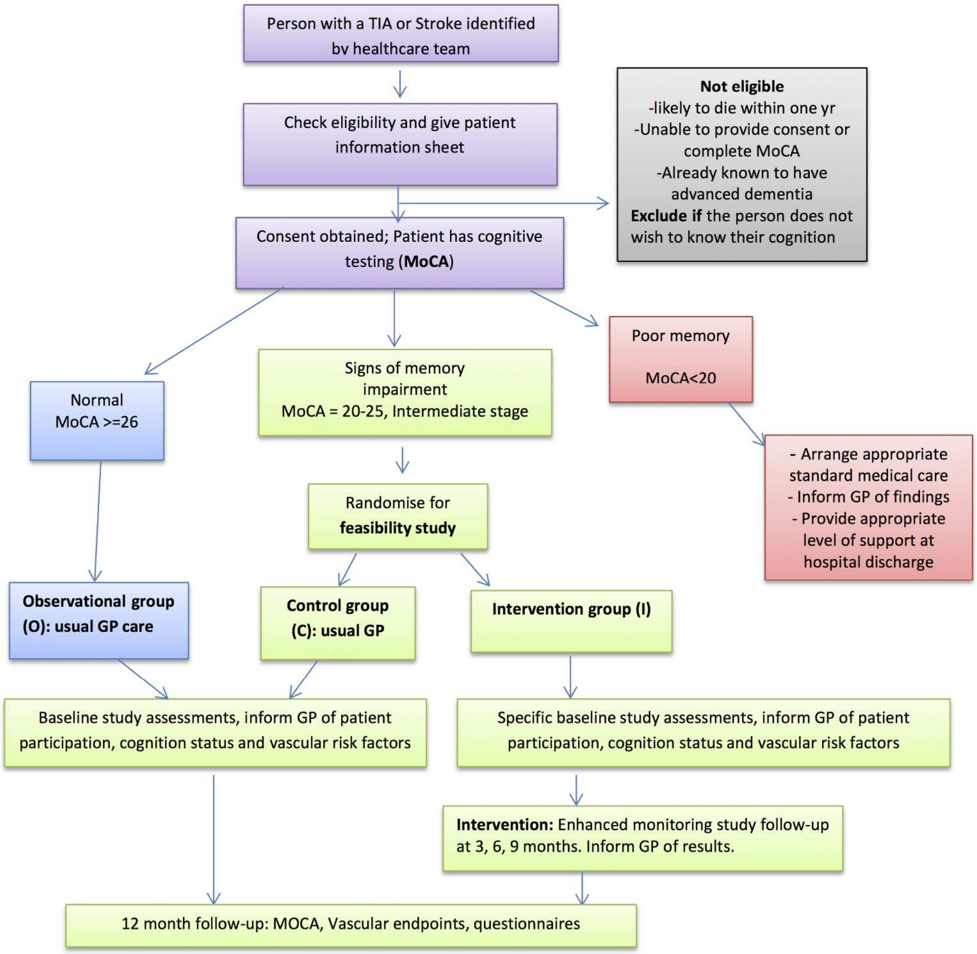

199x196mm (300 x 300 DPI)

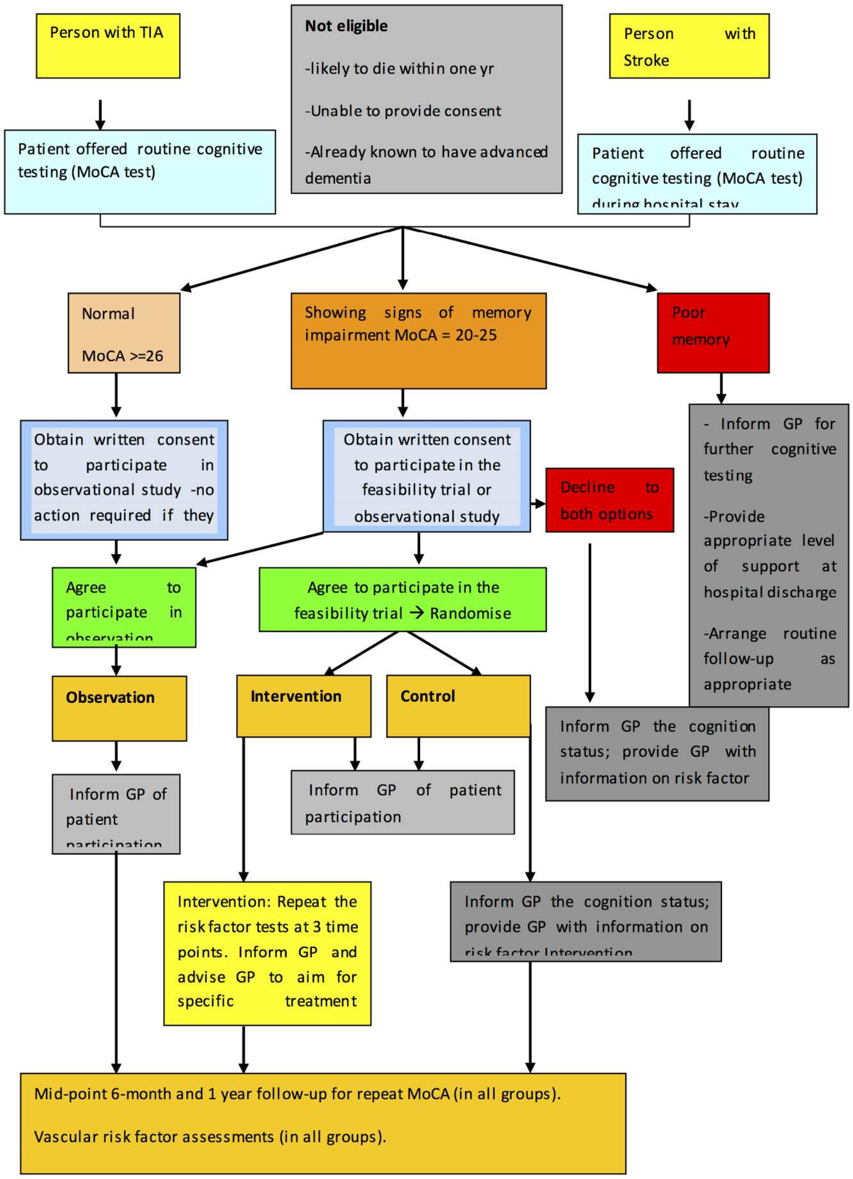

174x239mm (300 x 300 DPI)

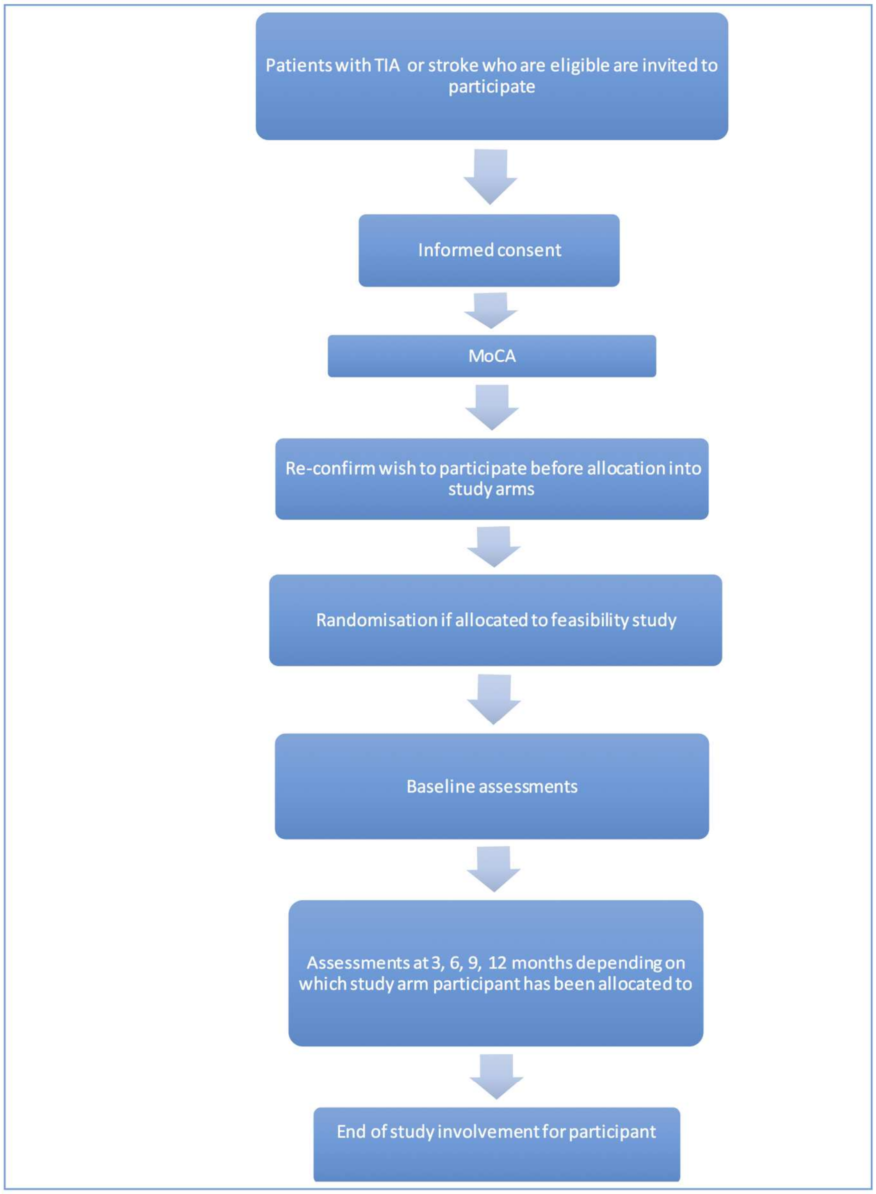

179x241mm (300 x 300 DPI)

Procedures	Visits		
	Screening	Baseline	12 months
**All Patients**			
Informed Consent	✓	✓	
Demographics		✓	
Medical history	✓		
Concomitant medications		✓	✓
Eligibility assessment	✓	✓	✓
Physical examination including VRFs		✓	✓
Blood sample for cholesterol, and where relevant, for warfarin and diabetes		✓	✓
Cognitive Assessment: MoCA	✓*		✓
Adverse events			✓
Quality of Life and Functional Assessment: EQ5D, DEMQOL, GDS, Bristol Activities of Daily Living, Morisky Medication Score		✓	✓
Resource use questionnaires		✓	✓
**Additional Procedures for Group C**			
24hr BP, beat to beat BP monitoring, Pulse wave velocity		✓	✓

*Unless already undertaken by AHP or TIA clinic team following the TIA/stroke

163x185mm (300 x 300 DPI)

	Baseline	**3 months**	**6 months**	**9 months**	12 months
Eligibility Assessment		✓	✓	✓	
Vascular Risk Factors		✓	✓	✓	
Blood sample for cholesterol, and where relevant, for warfarin and diabetes		✓	✓	✓	
24hr BP, beat to beat BP monitoring, Pulse wave velocity	✓				✓
Concomitant medications and adherence		✓	✓	✓	
Adverse Events		✓	✓	✓	

191x103mm (300 x 300 DPI)

review only

SPIRIT Checklist

SPIRIT 2013 Checklist: Recommended items to address in a clinical trial protocol and related documents*

Section/item	Item No	Description	Addressed on page number
**Administrative information**			
Title	1	Descriptive title identifying the study design, population, interventions, and, if applicable, trial acronym	_______1_____
Trial registration	2a	Trial identifier and registry name. If not yet registered, name of intended registry	_____4 & 6____
	2b	All items from the World Health Organization Trial Registration Data Set	______________
Protocol version	3	Date and version identifier	_______1______
Funding	4	Sources and types of financial, material, and other support	______14_____
Roles and responsibilities	5a	Names, affiliations, and roles of protocol contributors	______1-2_____
	5b	Name and contact information for the trial sponsor	_______1______
	5c	Role of study sponsor and funders, if any, in study design; collection, management, analysis, and interpretation of data; writing of the report; and the decision to submit the report for publication, including whether they will have ultimate authority over any of these activities	______N/A_____

For peer review only

	5d	Composition, roles, and responsibilities of the coordinating centre, steering committee, endpoint adjudication committee, data management team, and other individuals or groups overseeing the trial, if applicable (see Item 21a for data monitoring committee)	_____13______
**Introduction**			
Background and rationale	6a	Description of research question and justification for undertaking the trial, including summary of relevant studies (published and unpublished) examining benefits and harms for each intervention	___4-5______
	6b	Explanation for choice of comparators	____5_______
Objectives	7	Specific objectives or hypotheses	____5_______
Trial design	8	Description of trial design including type of trial (eg, parallel group, crossover, factorial, single group), allocation ratio, and framework (eg, superiority, equivalence, noninferiority, exploratory)	____5_______
**Methods: Participants, interventions, and outcomes**			
Study setting	9	Description of study settings (eg, community clinic, academic hospital) and list of countries where data will be collected. Reference to where list of study sites can be obtained	____5_______
Eligibility criteria	10	Inclusion and exclusion criteria for participants. If applicable, eligibility criteria for study centres and individuals who will perform the interventions (eg, surgeons, psychotherapists)	____6______
Interventions	11a	Interventions for each group with sufficient detail to allow replication, including how and when they will be administered	_8-9 & 10-11_

For peer review only

	11b	Criteria for discontinuing or modifying allocated interventions for a given trial participant (eg, drug dose change in response to harms, participant request, or improving/worsening disease)	_____N/A_____
	11c	Strategies to improve adherence to intervention protocols, and any procedures for monitoring adherence (eg, drug tablet return, laboratory tests)	_____N/A_____
	11d	Relevant concomitant care and interventions that are permitted or prohibited during the trial	_____N/A_____
Outcomes	12	Primary, secondary, and other outcomes, including the specific measurement variable (eg, systolic blood pressure), analysis metric (eg, change from baseline, final value, time to event), method of aggregation (eg, median, proportion), and time point for each outcome. Explanation of the clinical relevance of chosen efficacy and harm outcomes is strongly recommended	____11-13_____
Participant timeline	13	Time schedule of enrolment, interventions (including any run-ins and washouts), assessments, and visits for participants. A schematic diagram is highly recommended (see Figure)	___9-11______
Sample size	14	Estimated number of participants needed to achieve study objectives and how it was determined, including clinical and statistical assumptions supporting any sample size calculations	_____12______
Recruitment	15	Strategies for achieving adequate participant enrolment to reach target sample size	_____7_______

Methods: Assignment of interventions (for controlled trials)

Allocation:

Sequence generation	16a	Method of generating the allocation sequence (eg, computer-generated random numbers), and list of any factors for stratification. To reduce predictability of a random sequence, details of any planned restriction (eg, blocking) should be provided in a separate document that is unavailable to those who enrol participants or assign interventions	____9-10_____
Allocation concealment mechanism	16b	Mechanism of implementing the allocation sequence (eg, central telephone; sequentially numbered, opaque, sealed envelopes), describing any steps to conceal the sequence until interventions are assigned	____9-10_____
Implementation	16c	Who will generate the allocation sequence, who will enrol participants, and who will assign participants to interventions	____9-10_____
Blinding (masking)	17a	Who will be blinded after assignment to interventions (eg, trial participants, care providers, outcome assessors, data analysts), and how	_____N/A____
	17b	If blinded, circumstances under which unblinding is permissible, and procedure for revealing a participant's allocated intervention during the trial	_____N/A_____

Methods: Data collection, management, and analysis

Data collection methods	18a	Plans for assessment and collection of outcome, baseline, and other trial data, including any related processes to promote data quality (eg, duplicate measurements, training of assessors) and a description of study instruments (eg, questionnaires, laboratory tests) along with their reliability and validity, if known. Reference to where data collection forms can be found, if not in the protocol	_____8-11____
	18b	Plans to promote participant retention and complete follow-up, including list of any outcome data to be collected for participants who discontinue or deviate from intervention protocols	____11-12____

Data management	19	Plans for data entry, coding, security, and storage, including any related processes to promote data quality (eg, double data entry; range checks for data values). Reference to where details of data management procedures can be found, if not in the protocol	___11 & 13____
Statistical methods	20a	Statistical methods for analysing primary and secondary outcomes. Reference to where other details of the statistical analysis plan can be found, if not in the protocol	_____12-13____
	20b	Methods for any additional analyses (eg, subgroup and adjusted analyses)	_____12-13____
	20c	Definition of analysis population relating to protocol non-adherence (eg, as randomised analysis), and any statistical methods to handle missing data (eg, multiple imputation)	_____12-13____

Methods: Monitoring

Data monitoring	21a	Composition of data monitoring committee (DMC); summary of its role and reporting structure; statement of whether it is independent from the sponsor and competing interests; and reference to where further details about its charter can be found, if not in the protocol. Alternatively, an explanation of why a DMC is not needed	_____13_____
	21b	Description of any interim analyses and stopping guidelines, including who will have access to these interim results and make the final decision to terminate the trial	_____13______
Harms	22	Plans for collecting, assessing, reporting, and managing solicited and spontaneously reported adverse events and other unintended effects of trial interventions or trial conduct	_____9-10____
Auditing	23	Frequency and procedures for auditing trial conduct, if any, and whether the process will be independent from investigators and the sponsor	_____13______

Ethics and dissemination

Research ethics approval	24	Plans for seeking research ethics committee/institutional review board (REC/IRB) approval	______13______
Protocol amendments	25	Plans for communicating important protocol modifications (eg, changes to eligibility criteria, outcomes, analyses) to relevant parties (eg, investigators, REC/IRBs, trial participants, trial registries, journals, regulators)	______13______
Consent or assent	26a	Who will obtain informed consent or assent from potential trial participants or authorised surrogates, and how (see Item 32)	______7-8_____
	26b	Additional consent provisions for collection and use of participant data and biological specimens in ancillary studies, if applicable	______N/A_____
Confidentiality	27	How personal information about potential and enrolled participants will be collected, shared, and maintained in order to protect confidentiality before, during, and after the trial	______13______
Declaration of interests	28	Financial and other competing interests for principal investigators for the overall trial and each study site	______14______
Access to data	29	Statement of who will have access to the final trial dataset, and disclosure of contractual agreements that limit such access for investigators	_____________
Ancillary and post-trial care	30	Provisions, if any, for ancillary and post-trial care, and for compensation to those who suffer harm from trial participation	_____________
Dissemination policy	31a	Plans for investigators and sponsor to communicate trial results to participants, healthcare professionals, the public, and other relevant groups (eg, via publication, reporting in results databases, or other data sharing arrangements), including any publication restrictions	______3______
	31b	Authorship eligibility guidelines and any intended use of professional writers	_____________

For peer review only

	31c	Plans, if any, for granting public access to the full protocol, participant-level dataset, and statistical code	______N/A______
**Appendices**			
Informed consent materials	32	Model consent form and other related documentation given to participants and authorised surrogates	___17-18____
Biological specimens	33	Plans for collection, laboratory evaluation, and storage of biological specimens for genetic or molecular analysis in the current trial and for future use in ancillary studies, if applicable	______N/A______

For peer review only

BMJ Open

Protocol for a Feasibility Randomised Controlled Trial of Screening & Enhanced Risk Management for Vascular Event related Decline in Memory (SERVED Memory)

Journal:	*BMJ Open*
Manuscript ID	bmjopen-2017-017416.R1
Article Type:	Protocol
Date Submitted by the Author:	08-Sep-2017

Complete List of Authors:	Myint, Phyo; University of East Anglia, School of Medicine, Medical Sciences & Nutrition<br>Loke, Yoon; University of East Anglia, Norwich Medical School<br>Davison, William; University of East Anglia Norwich Medical School<br>Mattishent, Katharina; University of East Anglia, Norwich Medical School<br>Fox, Chris; Norwich Medical School, Department of Psychological Sciences<br>Fleetcroft, Robert; University of East Anglia<br>Turner, David; University of East Anglia, Public Health and Primary Care<br>Shepstone, Lee; University of East Anglia<br>Potter, John; University of East Anglia, Norwich Medical School; Norfolk and Norwich University Hospital, Stroke Research Group

**Primary Subject Heading**:	Cardiovascular medicine
Secondary Subject Heading:	Neurology, Geriatric medicine
Keywords:	Stroke < NEUROLOGY, Dementia < NEUROLOGY, VASCULAR MEDICINE, STROKE MEDICINE

SCHOLARONE™ Manuscripts

Protocol for a Feasibility Randomised Controlled Trial of Screening & Enhanced Risk Management for Vascular Event related Decline in Memory (SERVED Memory)

Correspondence to:

Professor Phyo Kyaw Myint

Room 4.013, Polwarth Building

Foresterhill, University of Aberdeen

Aberdeen, AB25 2ZD

UK

Tel: + 44 (0) 1224 437841

Fax: +44 (0) 1224 437911

Mail to: phyo.myint@abdn.ac.uk

Keywords: cognitive impairment, dementia, vascular dementia, stroke, TIA

Word count (excluding title page, refs, tables, figures): 3823 words

Authors:

Professor Phyo Kyaw Myint, Professor of Medicine of Old Age, University of Aberdeen, Aberdeen, UK

Professor Yoon K Loke, Professor of Medicine & Pharmacology, University of East Anglia, Norwich, UK

Dr William Davison, Clinical Research Fellow in Stroke Medicine, University of East Anglia, Norwich, UK

Dr Katharina Mattishent, Clinical Research Fellow in Older Peoples' Medicine, University of East Anglia, Norwich, UK

Professor George Christopher Fox, Professor of Psychiatry, University of East Anglia, Norwich, UK

Dr Robert Fleetcroft, General Practitioner and Honorary Senior Fellow, Norwich Medical School, Norwich, UK

Mr David Turner, Senior Researcher in Health Economics, Norwich Medical School, Norwich, UK

Professor Lee Shepstone, Professor of Medical Statistics, University of East Anglia, Norwich, UK

Professor John F Potter, Professor of Ageing & Stroke Medicine, University of East Anglia, Norwich, UK

Author Contributions:

For peer review only

P Myint, J Potter, Y Loke, G Fox, R Fleetcroft, D Turner and L Shepstone were involved in the original conception and design of the study. The original protocol was written by P Myint, J Potter, Y Loke and K Mattishent, and reviewed by the other authors listed above.

W Davison is part of the research team involved in recruitment and data acquisition and was responsible for revising the protocol for submission to BMJ Open, with input from all other contributing authors. This manuscript has been adapted from protocol version 3.3 dated 3/5/16. The final manuscript has been reviewed by all authors and approved for submission/publication.

For peer review only

ABSTRACT

Introduction: Stroke is a leading cause of death and disability. The development of dementia after stroke is common. Vascular risk factors (VRF) which contribute to stroke risk can also contribute to cognitive decline, especially in vascular dementia (VaD). There is no established treatment for VaD, therefore strategies for prevention could have major health resource implications. This study was designed to assess whether patients with early cognitive decline after stroke/transient ischaemic attack (TIA) can be easily identified and whether target driven VRF management can prevent progression to dementia.

The primary objective is to establish the feasibility of recruitment and retention of patients with early cognitive decline to a randomised controlled trial of enhanced VRF management. Secondary objectives include (a) to determine the potential clinical benefit of the intervention; (b) to estimate the sample size for a future definitive multi-centre randomised controlled trial; (c) to inform a future economic evaluation; (d) to explore the link between VRF control and the incidence of cognitive impairment on longitudinal follow-up in a UK population after stroke/TIA with current routine management.

Methods: 100 patients with cognitive decline post-stroke/TIA will be recruited from stroke services at the Norfolk and Norwich University Hospital (NNUH). After collection of baseline data they will be randomised to intervention (3 monthly follow-up with enhanced management) or control (treatment as usual by the General Practitioner (GP)). At 12 months outcomes (repeat cognitive testing, VRF assessment) will be assessed. A further 100 patients without cognitive decline will be recruited to a parallel observational group from the same site. At 12 months they will have repeat cognitive testing.

Ethics and dissemination: Ethical approval has been granted in England. Dissemination is planned via publication in peer-reviewed medical journals and presentation at relevant conferences.

Registration details: International standard randomised controlled trial number (ISRCTN) 42688361.

For peer review only

Strengths and limitations of this study:

- The protocol utilises a validated cognitive screening test which is sensitive and specific for the detection of mild cognitive impairment as well as dementia.
- Data will be collected on a range of VRF.
- The study is open-label, but repeat cognitive testing will be completed by a member of the research team who is blinded to allocation and baseline cognitive status.
- The chosen follow-up period of 12 months may limit our ability to detect changes in cognition.

INTRODUCTION

Background

Stroke is one of the leading causes of death and disability [1] and current demographic trends suggest that the total numbers of people with a stroke will rise due to the ageing population [2]. Cognitive decline after stroke poses a significant problem considering that up to 30% of patients may potentially develop dementia as early as 3-months after their cerebrovascular event [3]. Stroke may unmask previously unrecognised cognitive impairment [4, 5], or may trigger new cognitive decline due to VaD, Alzheimer's disease, or mixed pathology [6, 7].

The World Alzheimer Report emphasised the benefit of early diagnosis with future savings from delayed institutionalisation, and care costs across the disease course [8]. Similarly the UK Government has identified the timely diagnosis of dementia in primary care as a priority [9]. An effective strategy in preventing VaD could have major resource implications - with at least 1 in 5 dementia cases having a VaD element and dementia costing the UK economy £23 billion per year [10]. Most importantly, *"how best to improve cognition after stroke"* was reported to be the highest priority research topic in a survey of patients with stroke [11]. Identifying patients who have signs of early cognitive decline after stroke or TIA could provide a window of opportunity for saving resources and improving patient outcomes if further cognitive decline could be prevented [6].

It is reported that dementia is common after stroke and TIA [3, 4, 12, 13]. Reported rates range from 7.4% up to 41.3% with the variance mostly dependent on the mix of the cohort (e.g. rates are higher in secondary care cohorts and those with higher rates of recurrent stroke) [14]. Our previous work shows that the risk of developing cognitive impairment appears to be greater in people with higher numbers of VRF [4] and other work suggests that the presence of cardiovascular risk factors

For peer review only

increases the risk of early cognitive decline progressing to dementia [15]. Improved control of VRF leading to enhanced secondary stroke prevention may therefore help to prevent further cognitive decline after stroke/TIA in high-risk patients with evidence of early cognitive impairment.

Rationale for the Study

Observational evidence indicates that both VaD and Alzheimer's dementia may have risk factors in common with stroke, namely VRF such as high blood pressure (BP) and diabetes [16, 17]. Despite this, whether intervening to control these risk factors can prevent dementia remains unclear [16, 18]. Firstly, trials of antihypertensive therapy have been inconsistent. However, they may have been limited by high rates of treatment in placebo groups, high dropout rates, and short follow-up [18]. Of note, a large trial which recruited patients with stroke/TIA (PROGRESS) did demonstrate reduced cognitive decline but not dementia with treatment [16, 18]. Furthermore, meta-analysis of placebo controlled trials suggests that antihypertensive therapy reduces the risk of dementia [19]. Secondly, two randomised controlled trials have assessed the use of statins and found no benefit on cognition despite reduction in cholesterol levels [20]. Thirdly, in the ADVANCE study intensive blood glucose control in type 2 diabetics successfully reduced microvascular complications, but did not reduce rates of dementia [18]. Finally, whether anticoagulation for atrial fibrillation (AF) can prevent cognitive decline is, at present, not addressed by the available evidence [16, 17]. In spite of this uncertainty there is evidence, as alluded to earlier, that recurrent stroke is an important factor in post-stroke dementia [14]. Given that treating VRF is beneficial for secondary stroke prevention, it therefore remains plausible that this could also have an impact on cognitive decline post-stroke and further research is justified. Evidence to support this comes from a randomised controlled trial in Germany which demonstrated a significant reduction in the need for long-term care in older adults following an intervention involving systematic identification and evidence-based treatment of cardiovascular risk factors [21]. Although two trials similar to ours have investigated the use of an intervention targeted at controlling VRF for preventing cognitive decline after stroke and neither demonstrated a benefit of intervention at 12 months [22, 23], a key difference with our study is that we will be targeting patients who already have signs of cognitive impairment at baseline and therefore are at higher risk of further decline.

Routine cognitive testing using validated measures which are simple and quick, such as the Montreal Cognitive Assessment (MoCA), which are shown to be sensitive and specific in detecting vascular related cognition can identify those who are at risk of developing decline [24-26]. We believe therefore, that detection of early cognitive decline in stroke and TIA is feasible at the time of

For peer review only

diagnosis in secondary care and we propose that enhanced (target driven) VRF control is clinically effective, cost-effective and safe.

The reported incidence of dementia post-stroke is variable, with the highest rates being over 40% [7, 14]. Reporting differences are strongly attributable to variation in the cohorts studied [14]. There is also some evidence to suggest that cognitive decline after TIA or minor stroke (defined as National Institute of Health Stroke Scale <3) may be transient [27]. However, there is a lack of data based on the current UK population. This study will therefore incorporate a parallel observational arm with a view to generating relevant epidemiological data in order to provide better insight regarding cognition after stroke/TIA in this patient population.

Study objectives

The primary objective of the study is to determine the feasibility of randomising patients who have signs of early cognitive decline, but no dementia, into routine risk factor management or enhanced risk factor management by their GP, and to assess adherence to the proposed intervention by enrolled participants.

The secondary objectives are:

(a) to determine the potential clinical benefit of enhanced control of VRF in preventing progression of cognitive decline and the development of dementia post-stroke/TIA;

(b) to assess indicative cost-effectiveness of this intervention;

(c) to estimate the sample size for a future definitive multi-centre randomised controlled trial;

(d) to identify any adverse events due to the intervention, including rates of recurrent stroke/TIA;

(e) to explore the incidence of cognitive impairment on longitudinal follow-up in a UK population after stroke/TIA with current routine risk factor management.

METHODS AND ANALYSIS

Study overview

This study is a single-centre, open-label parallel group study to determine the feasibility of conducting a randomised controlled trial in an NHS setting on patients following stroke or TIA who have early cognitive decline. The aim is to target risk factors more intensively through enhanced monitoring and control of VRF compared to usual care. We wish to estimate the potential clinical

For peer review only

impact and cost-effectiveness of this intervention, and the sample size for a future multicentre definitive study in an NHS setting.

There is a parallel observational study arm for patients with no evidence of cognitive decline who will have their VRF and cognitive function assessed at follow-up. The objective of the parallel observational study is to better understand the link between VRF, their control and the development of cognitive decline after a cerebrovascular event. Combining the control arm of the feasibility trial and observational cohort will provide further information on these links, including a realistic estimate of the magnitude of effect of enhanced risk factor management in planning a future trial. A summary of the study design is provided in **Figure 1.** Recruitment commenced in November 2015, was completed in July 2017, and final follow-up data collection will be in July 2018. The study has been registered on 16th April 2015: International standard randomised controlled trial number (ISRCTN) 42688361.

Trial Participants

All adult patients with confirmed stroke (first/recurrent) or TIA, identified within 8 weeks of diagnosis will be considered for the trial.

Inclusion Criteria

- Participant is willing and able to give informed consent for participation;
- Male or female, aged 18 years or above;
- Diagnosed clinically and radiologically with stroke (infarct or haemorrhage) or TIA.

Exclusion Criteria

The participant may not enter the trial if ANY of the following apply:

- Established Dementia;
- Life expectancy <1 year;
- Co-morbidities that adversely affect their ability to accurately complete the MoCA;
- Patients who do not wish to know about their cognition.

Identification of participants

Eligible patients will be given a detailed Patient Information Sheet (PIS) and Consent Form for consideration. After 24 hours the study team will contact the patient again and those who agree to participate will provide written informed consent and undergo a simple and validated cognitive screening test (MoCA), unless this has already been carried out by the clinical team as part of their

For peer review only

routine care, whereupon that score can be used for study purposes. If a MoCA has been administered by the TIA clinic team the patient will be given a PIS in clinic and be followed up by phone to discuss entry into the study. Patients who have previously attended stroke services may be screened retrospectively from the Capture TIA/Stroke hospital database and followed up by phone. Those who have a MoCA score ≥26 and who verbally consent to be contacted about the study are sent a study summary sheet, a study invitation letter and consent form. If they wish to participate the completed consent form will be returned and countersigned by a delegated member of the study team. Those who do not have a MoCA score following their stroke or TIA are invited for an appointment to give consent to take part in the study and carry out the MoCA. The patient will be enrolled into the appropriate arm of the study depending on the MoCA score.

Assessing capacity and obtaining informed consent

The participant must personally sign and date the latest approved version of the Informed Consent Form. This will then be countersigned by a delegated member of the research team before any trial specific procedures are performed. Written and verbal versions of the PIS will be presented to the participants detailing the trial rationale; participant involvement and responsibilities; the implications and constraints of the protocol; safeguards; and processing of blood tests. It will be clearly stated that the participant is free to withdraw from the trial at any time for any reason without prejudice to future care, and with no obligation to give the reason for withdrawal. The member of the research team who takes consent will be delegated to do so, be familiar with the study and be suitably qualified to obtain consent for research purposes. A copy of the signed informed consent will be given to the participant, and another will be stored at NNUH Clinical Research Trials Unit (CRTU). The original signed form will be retained in the patient's medical records.

Allocation into Study Arms

Allocation is based on the patient's MoCA score, interpreted in the following manner:

- Score ≥26 indicates normal cognition, with this score being chosen to maximise the sensitivity of the test for detecting early cognitive decline.
- Score 20-25 suggests early cognitive decline [24, 25].
- Score ≤17 suggests possibility of dementia [28].

Normal cognition (MoCA score ≥26)

For peer review only

Patients with normal cognition will be informed of their result and will continue to receive usual care by their clinicians. They will be asked to confirm their willingness to continue to participate in the observational study **(GROUP O)** and will be followed-up at 12 months.

Intermediate stage (MoCA score 20-25)

Patients will be informed of their result and asked to confirm their willingness to continue to participate in the feasibility trial. They will be randomised into one of two groups; control arm **(GROUP C)** or intervention arm **(GROUP I)**. Randomisation will be based on computer generated blocked randomisation managed by the Norwich CRTU.

Patients in **GROUP C** will receive usual care by their clinicians and will be followed-up at 12 months.

Patients in **GROUP I** will undergo enhanced VRF management through assessment by the study team at 3, 6, and 9 months. Specific aims will be set for each modifiable risk factor and the GP will be informed about these targets and the results of each visit.

Greater degree of cognitive decline (MoCA <20)

In view of the greater extent of cognitive impairment, continued participation in the study is not suitable for these patients. They will be referred to specialist services where relevant, and their GP will be informed.

Interventions to be measured

Blood pressure

Existing evidence demonstrates that lowering of BP consistently and continuously reduces cardiovascular risk. This is supported by the Royal College of Physicians (RCP) guidelines' aim for BP 140/90 mmHg, with an ideal target BP <130/80 mmHg for secondary prevention [29]. The effect of intervention on BP reduction using 24 hour BP measurement at the beginning and end of the study will be examined. This will be recorded using a SpaceLabs 90207 monitor programmed to measure BP at 20 minute intervals during the daytime (0700-2200) and hourly overnight (2200-0700).

Plasma Lipids

The link between total cholesterol and dementia is controversial, but from a cardiovascular risk factor point of view lowering cholesterol by using statins improves stroke secondary prevention. Although post-stroke dementia can be of VaD type, Alzheimer's dementia, or mixed pathology, lowering cholesterol should contribute to a reduced risk of post-stroke dementia where the mechanism is one of VaD. Therefore, the cholesterol aim has been chosen as one of the "treat to target" interventions. The total cholesterol aim will be <4.0 mmol/L which is in line with the RCP guidelines which were current at the trial inception [29].

Atrial fibrillation

For peer review only

It has been shown that people with stroke and AF are more likely to be subsequently diagnosed with dementia [3, 5]. An intervention rate aim of 60-80 beats per minute has been chosen for patients with AF; those on warfarin will aim for an INR between 2.5 and 3 to maintain levels in the therapeutic window. There are no specific drug monitoring targets for other anticoagulants.
10 minutes of continuous beat-to-beat BP measurement will be carried out at baseline and the final follow-up using a Finometer device. This data can be used to assess heart rate variability as well as blood pressure variability.

Blood glucose

Diabetes mellitus is associated with both micro and macrovascular disease, and hence carotid artery disease which is a preventable risk factor for stroke. It has been well documented that poor glucose control (assessed using HbA1C) predicts stroke risk [30]. Therefore good diabetes control may prevent further cardiovascular risk and be associated with added benefit to future cognitive status. The aim is for HbA1C of 48-53 mmol/mol (or 6.5-7%).

The patient's GP will be informed by letter of the results that have been recorded during the research study. All patients will receive standard lifestyle advice relating to diet and weight, smoking, and alcohol consumption.

Ordering of assessments (Table 1)

The following assessments will be carried out on all participants at baseline:

- Eligibility assessment and informed consent;
- MoCA;
- Demographics, including age, gender, body mass index, smoking status, alcohol consumption exercise habits;
- Assessment of past medical history, including VRF;
- Record concomitant medications.

In addition, participants in group C and I will complete at baseline:

- EQ5D (a generic health-related quality of life questionnaire), DEMQOL (a dementia-specific QoL questionnaire), GDS (Geriatric Depression Scale questionnaire), Bristol Activities of Daily Living questionnaire and Morisky Medication Adherence Score;
- Resource use questionnaires;
- BP variability measures including 10 minutes of continuous beat-to-beat BP recording and 24 hour BP monitoring;

For peer review only

- Pulse wave velocity (PWV) measurements (which reflect arterial stiffness).

Participants in group I will be seen at 3, 6 and 9 months at which time they will have assessment of their VRF and data collection for adverse events, including recurrent stroke/TIA.

All participants will be followed-up at 12 months at which time they will have:

- MoCA;
- Assessment of VRF;
- Recording of concomitant medications;
- Data collection for adverse events, including recurrent stroke/TIA.

In addition, participants in group C and I will complete at 12 months:

- EQ5D, DEMQOL, GDS, Bristol Activities of Daily Living questionnaire and Morisky Medication Adherence Score;
- Resource use questionnaires;
- BP variability measures;
- PWV measurements.

Table 1: Summary of study procedures

**Procedures for all participants**	**Visits**					
	**Screening**	**Baseline**	**3 months**	**6 months**	**9 months**	**12 months**
Eligibility assessment	✓	✓				✓
Informed consent	✓	✓				
Montreal Cognitive Assessment	✓					✓
Medical history	✓					
Demographics		✓				
Concomitant medications		✓				✓
Physical examination including VRFs		✓				✓
Blood sample for cholesterol +/- INR and blood glucose/HbA1C		✓				✓
Adverse events						✓
**Additional procedures for participants in Group C**						
24 hour BP measurement		✓				✓
Beat to beat BP		✓				✓

For peer review only

measurement						
Pulse wave velocity measurement		✓				✓
Quality of life and functional assessment*		✓				✓
Resource use questionnaires		✓				✓
**Additional procedures for participants in Group I**						
Eligibility assessment			✓	✓	✓	
Assessment of VRF’s			✓	✓	✓	
Blood sample for cholesterol +/- INR and blood glucose/HbA1C			✓	✓	✓	
Concomitant medications and adherence			✓	✓	✓	
Quality of life and functional assessment*		✓				✓
Resource use questionnaires		✓				✓
24 hour BP measurement		✓				✓
Beat to beat BP measurement		✓				✓
Pulse wave velocity measurement		✓				✓
Adverse events			✓	✓	✓	

* Includes EQ5D, DEMQOL, Geriatric Depression Scale, Bristol Activities of Daily Living, Morisky Medication Score.

For peer review only

Outcome measurements

1. Primary outcome measure:

Recruitment and retention rates at 12 months from the screening and study management logs.

2. Secondary outcome measures:

(a) difference in mean change in MoCA score between groups C and I at 12 months;

(b) proportions of participants in each group whose vascular risk factors are controlled at each time point;

(c) frequency of adverse events in each group;

(d) indicative incremental cost per MoCA point and QALY gained by the intervention;

(e) mean change in MoCA score in group O related to number of VRF and proportion of participants whose VRF are controlled at baseline and outcome.

Sample size calculation

As this is a feasibility study a formal sample size calculation has not been performed. The duration and sample size of the study are based on the estimated prevalence rate of cognitive impairment at diagnosis (around 30%) [4], incidence of dementia after the event (~30% in 3 months) [3], estimated screening and recruitment rates. The aim is to include a minimum of 100 patients in the feasibility study (50 per group) and another 100 patients in the observational study.

Data analysis plan

Primary objective:

- Proportion of participants with MoCA score 20-25 who consent to join the trial;
- Adherence to follow-up, including rates of withdrawal and loss to follow-up;
- Number of risk factors that need to be targeted in these patients administering the client service receipt inventory (CSRI) and Quality of Life questionnaires. Originally designed for costing psychiatric interventions, the CSRI [31] has been used as the core resource use measurement tool for a wide variety of interventions. It requires adapting to fit each study question therefore the feasibility study will allow testing and revising of the questionnaire in preparation for the full study.

Secondary objectives:

- Rates of control of VRF at baseline and outcome in each group;
- Proportion of participants in group I achieving VRF targets at 3, 6 and 9 months for each risk factor;
- Difference in mean change in MoCA score between groups C and I. The between group comparison will be based on a general linear model with group as a fixed effect and including any prognostic variables at baseline for which there is a between group disparity. A 95% confidence interval for the difference in means will be constructed to give an idea of the likely magnitude of benefit from the intervention;
- Indicative incremental cost per point gained in MoCA, DEMQOL and per QALY gained between Group C and I. Data will be analysed in terms of costs and effects for the two groups. We will analyse key drivers of costs and examine the potential of this intervention to be cost-effective.
- Change in MoCA score between baseline and 12 months in GROUP O participants, and in groups O and C combined;
- Difference in adverse event rates between groups;
- Difference in mean BP between Groups C and I;

For peer review only

- Difference in BP (systolic and diastolic) variability between Groups C and I;
- Difference in PWV between Groups C and I.

ETHICS AND DISSEMINATION

This study was granted ethical approval in England (East of England Cambridge East Research Ethics Committee, REC 15/EE/0061). Study oversight will be conducted through regular meetings of a Trial Steering Committee and a separate Safety Committee, both of which will include independent representatives. If it is felt that the risk to participants is significant or unacceptable the Safety Committee can recommend to early termination of the trial.

Data will be collected and handled in line with sponsor and Norwich CRTU procedures and NHS Trust policies. Electronic data will be anonymised and all data will be kept under secure conditions. Professor Potter will act as data custodian.

Dissemination of the study results is planned via publication in peer-reviewed medical journals and presentation at relevant scientific conferences. Any reporting will adhere to the CONSORT statement extension for pilot and feasibility trials. We do not intend to employ professional writers.

Competing Interests: none declared.

This work was supported by the National Institute for Health Research (NIHR) under its Research for Patient Benefit Programme (RfPB), grant number DRF-2013-06-115. The views expressed here are those of the author(s) and not necessarily those of the NHS, the NIHR or the Department of Health.

The study is sponsored by Research and Development at NNUH. The sponsor has not had a role in the design or implementation of the study, nor the writing of this protocol.
Contact details for the sponsor:
Laura Harper (Research Study and Recruitment Facilitator)
Norfolk and Norwich University Hospitals NHS Foundation Trust
Colney Lane
Norwich, NR4 7UY
Tel: +44 (0) 1603 287408
Fax: +44 (0) 1603 288314

For peer review only

Mail to: laura.harper@nnuh.nhs.uk

Figures included:

Figure 1: Summary of the study design depicting the flow of participants through the study. Steps detailed include the identification and recruitment of participants, allocation and randomisation into the study arms based on MoCA score, and the timing of intervention and follow-up visits.

References

1. The Stroke Association. *State of the nation: stroke statistics January 2017*. 2017 [cited 2017 17th July 2017]; Available from: https://www.stroke.org.uk/resources/state-nation-stroke-statistics.
2. Myint, P.K., et al., *Risk factors for first-ever stroke in the EPIC-Norfolk prospective population-based study.* Eur J Cardiovasc Prev Rehabil, 2008. **15**(6): p. 663-9.
3. Kwok, C.S., et al., *Atrial fibrillation and incidence of dementia: a systematic review and meta-analysis.* Neurology, 2011. **76**(10): p. 914-22.
4. Guyomard, V., et al., *Transient ischaemic attack, vascular risk factors and cognitive impairment: a case-controlled study.* Age Ageing, 2011. **40**(5): p. 641-4.
5. Barba, R., et al., *Poststroke dementia : clinical features and risk factors.* Stroke, 2000. **31**(7): p. 1494-501.
6. Brainin, M., et al., *Post-stroke cognitive decline: an update and perspectives for clinical research.* Eur J Neurol, 2015. **22**(2): p. 229-38, e13-6.
7. Leys, D., et al., *Poststroke dementia.* Lancet Neurol, 2005. **4**(11): p. 752-9.
8. Wimo, A. and M. Prince. *World Alzheimer Report 2010: The Global Economic Impact of Dementia*. 2010 21st August 2017]; Available from: https://www.alz.co.uk/research/world-report-2010.
9. National Audit Office. *Reducing Brain Damage: Faster access to better stroke care*. 2005 [cited 2017 17th July 2017]; Available from: https://www.nao.org.uk/report/department-of-health-reducing-brain-damage-faster-access-to-better-stroke-care/.
10. Luengo-Fernandez, R., J. Leal, and A. Gray. *Dementia 2010 - the prevalence, economic cost and research funding of dementia compared with other major diseases*. 2010 21st August 2017]; Available from: https://www.cddft.nhs.uk/media/185806/dementia%202010%20exec%20summary.pdf.
11. Pollock, A., et al., *Top ten research priorities relating to life after stroke.* Lancet Neurol, 2012. **11**(3): p. 209.
12. Allan, L.M., et al., *Long term incidence of dementia, predictors of mortality and pathological diagnosis in older stroke survivors.* Brain, 2011. **134**(Pt 12): p. 3716-27.
13. van Rooij, F.G., et al., *Persistent cognitive impairment after transient ischemic attack.* Stroke, 2014. **45**(8): p. 2270-4.
14. Pendlebury, S.T. and P.M. Rothwell, *Prevalence, incidence, and factors associated with pre-stroke and post-stroke dementia: a systematic review and meta-analysis.* Lancet Neurol, 2009. **8**(11): p. 1006-18.
15. Ettorre, E., et al., *Role of cardiovascular risk factors (CRF) in the patients with mild cognitive impairment (MCI).* Arch Gerontol Geriatr, 2012. **54**(2): p. 330-2.
16. Dichgans, M. and V. Zietemann, *Prevention of vascular cognitive impairment.* Stroke, 2012. **43**(11): p. 3137-46.

For peer review only

17. Duron, E. and O. Hanon, *Vascular risk factors, cognitive decline, and dementia.* Vasc Health Risk Manag, 2008. **4**(2): p. 363-81.

18. Ligthart, S.A., et al., *Treatment of cardiovascular risk factors to prevent cognitive decline and dementia: a systematic review.* Vasc Health Risk Manag, 2010. **6**: p. 775-85.

19. Peters, R., et al., *Incident dementia and blood pressure lowering in the Hypertension in the Very Elderly Trial cognitive function assessment (HYVET-COG): a double-blind, placebo controlled trial.* Lancet Neurol, 2008. **7**(8): p. 683-9.

20. McGuinness, B., et al., *Statins for the prevention of dementia.* Cochrane Database Syst Rev, 2016(1): p. CD003160.

21. Bickel, H., et al., *Reduction of Long-Term Care Dependence After an 8-Year Primary Care Prevention Program for Stroke and Dementia: The INVADE Trial.* J Am Heart Assoc, 2012. **1**(4): p. e000786.

22. Ihle-Hansen, H., et al., *Multifactorial vascular risk factor intervention to prevent cognitive impairment after stroke and TIA: a 12-month randomized controlled trial.* Int J Stroke, 2014. **9**(7): p. 932-8.

23. Matz, K., et al., *Multidomain Lifestyle Interventions for the Prevention of Cognitive Decline After Ischemic Stroke: Randomized Trial.* Stroke, 2015. **46**(10): p. 2874-80.

24. Nasreddine, Z.S., et al., *The Montreal Cognitive Assessment, MoCA: a brief screening tool for mild cognitive impairment.* J Am Geriatr Soc, 2005. **53**(4): p. 695-9.

25. Pendlebury, S.T., et al., *MoCA, ACE-R, and MMSE versus the National Institute of Neurological Disorders and Stroke-Canadian Stroke Network Vascular Cognitive Impairment Harmonization Standards Neuropsychological Battery after TIA and stroke.* Stroke, 2012. **43**(2): p. 464-9.

26. Webb, A.J.S., et al., *Validation of the Montreal Cognitive Assessment Versus Mini-Mental State Examination Against Hypertension and Hypertensive Arteriopathy After Transient Ischemic Attack or Minor Stroke.* Stroke, 2014. **45**(11): p. 3337-3342.

27. Sivakumar, L., et al., *Serial montreal cognitive assessments demonstrate reversible cognitive impairment in patients with acute transient ischemic attack and minor stroke.* Stroke, 2014. **45**(6): p. 1709-15.

28. Freitas, S., et al., *Montreal Cognitive Assessment (MoCA): validation study for vascular dementia.* J Int Neuropsychol Soc, 2012. **18**(6): p. 1031-40.

29. Intercollegiate Stroke Working Party. *National clinical guideline for stroke 4th ed.* 2012 [cited 2017 17th July 2017]; Available from: https://www.strokeaudit.org/Guideline/Historical-Guideline.aspx.

30. Myint, P.K., et al., *Glycated hemoglobin and risk of stroke in people without known diabetes in the European Prospective Investigation into Cancer (EPIC)-Norfolk prospective population study: a threshold relationship?* Stroke, 2007. **38**(2): p. 271-5.

31. Beecham, J. and M. Knapp, *Costing Psychiatric Interventions*, in *Measuring Mental Health Needs*, G. Thornicroft, Editor. 2001, Gaskell. p. 200-224.

For peer review only

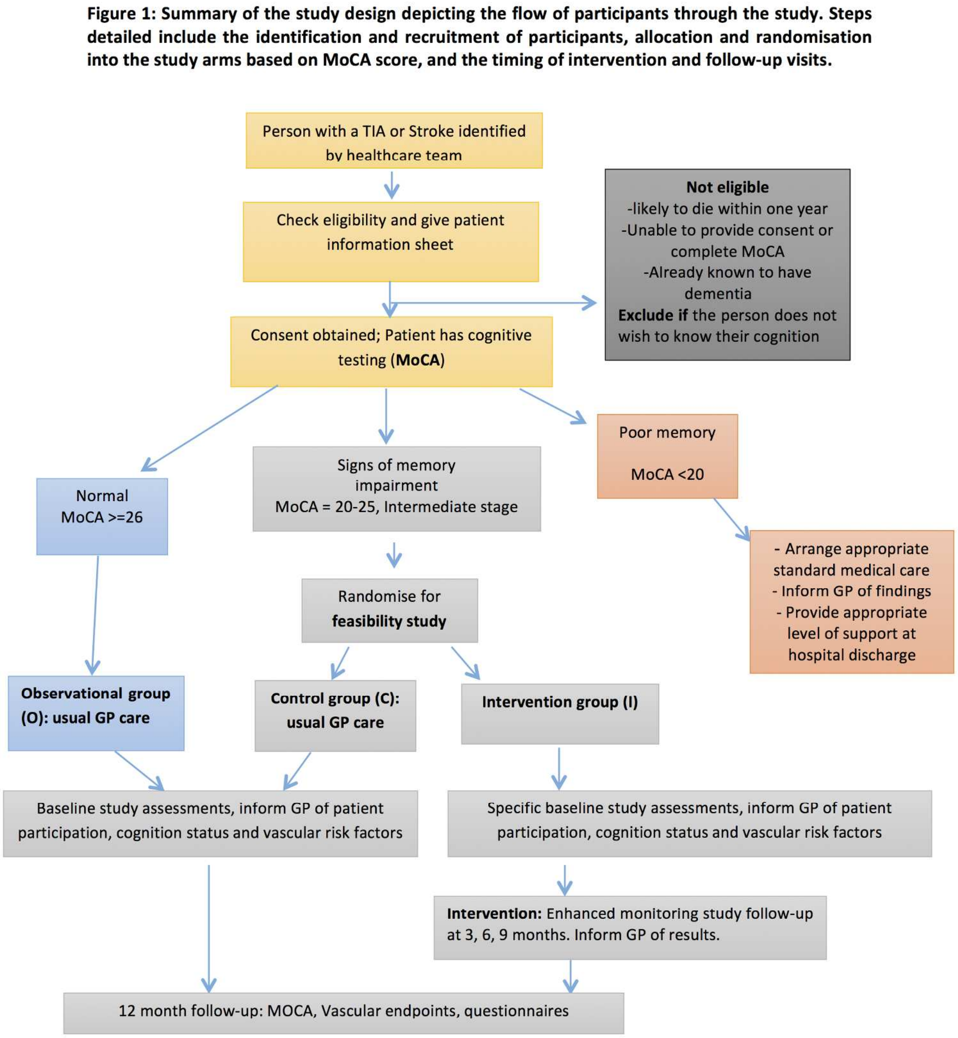

Figure 1: Summary of the study design depicting the flow of participants through the study. Steps detailed include the identification and recruitment of participants, allocation and randomisation into the study arms based on MoCA score, and the timing of intervention and follow-up visits.

203x238mm (300 x 300 DPI)

SPIRIT Checklist

SPIRIT 2013 Checklist: Recommended items to address in a clinical trial protocol and related documents*

Section/item	Item No	Description	Addressed on page number
**Administrative information**			
Title	1	Descriptive title identifying the study design, population, interventions, and, if applicable, trial acronym	_______1_____
Trial registration	2a	Trial identifier and registry name. If not yet registered, name of intended registry	____3 & 6____
	2b	All items from the World Health Organization Trial Registration Data Set	___Present__
Protocol version	3	Date and version identifier	_______2______
Funding	4	Sources and types of financial, material, and other support	_____14_____
Roles and responsibilities	5a	Names, affiliations, and roles of protocol contributors	_____1-2_____
	5b	Name and contact information for the trial sponsor	_____14_____
	5c	Role of study sponsor and funders, if any, in study design; collection, management, analysis, and interpretation of data; writing of the report; and the decision to submit the report for publication, including whether they will have ultimate authority over any of these activities	_____14_____

For peer review only

	5d	Composition, roles, and responsibilities of the coordinating centre, steering committee, endpoint adjudication committee, data management team, and other individuals or groups overseeing the trial, if applicable (see Item 21a for data monitoring committee)	_____14______

Introduction

Background and rationale	6a	Description of research question and justification for undertaking the trial, including summary of relevant studies (published and unpublished) examining benefits and harms for each intervention	___4-6______
	6b	Explanation for choice of comparators	____6-7_______
Objectives	7	Specific objectives or hypotheses	____6_______
Trial design	8	Description of trial design including type of trial (eg, parallel group, crossover, factorial, single group), allocation ratio, and framework (eg, superiority, equivalence, noninferiority, exploratory)	____6-7_______

Methods: Participants, interventions, and outcomes

Study setting	9	Description of study settings (eg, community clinic, academic hospital) and list of countries where data will be collected. Reference to where list of study sites can be obtained	____6-7_______
Eligibility criteria	10	Inclusion and exclusion criteria for participants. If applicable, eligibility criteria for study centres and individuals who will perform the interventions (eg, surgeons, psychotherapists)	____7______
Interventions	11a	Interventions for each group with sufficient detail to allow replication, including how and when they will be administered	_8-11 & Table 1_

For peer review only

	11b	Criteria for discontinuing or modifying allocated interventions for a given trial participant (eg, drug dose change in response to harms, participant request, or improving/worsening disease)	_____N/A_____
	11c	Strategies to improve adherence to intervention protocols, and any procedures for monitoring adherence (eg, drug tablet return, laboratory tests)	_____N/A_____
	11d	Relevant concomitant care and interventions that are permitted or prohibited during the trial	_____N/A_____
Outcomes	12	Primary, secondary, and other outcomes, including the specific measurement variable (eg, systolic blood pressure), analysis metric (eg, change from baseline, final value, time to event), method of aggregation (eg, median, proportion), and time point for each outcome. Explanation of the clinical relevance of chosen efficacy and harm outcomes is strongly recommended	____12_____
Participant timeline	13	Time schedule of enrolment, interventions (including any run-ins and washouts), assessments, and visits for participants. A schematic diagram is highly recommended (see Figure)	___9-11______
Sample size	14	Estimated number of participants needed to achieve study objectives and how it was determined, including clinical and statistical assumptions supporting any sample size calculations	____12-13___
Recruitment	15	Strategies for achieving adequate participant enrolment to reach target sample size	_____7-8_____

Methods: Assignment of interventions (for controlled trials)

Allocation:

Sequence generation	16a	Method of generating the allocation sequence (eg, computer-generated random numbers), and list of any factors for stratification. To reduce predictability of a random sequence, details of any planned restriction (eg, blocking) should be provided in a separate document that is unavailable to those who enrol participants or assign interventions	____8-9_____
Allocation concealment mechanism	16b	Mechanism of implementing the allocation sequence (eg, central telephone; sequentially numbered, opaque, sealed envelopes), describing any steps to conceal the sequence until interventions are assigned	____8-9_____
Implementation	16c	Who will generate the allocation sequence, who will enrol participants, and who will assign participants to interventions	____8-9_____
Blinding (masking)	17a	Who will be blinded after assignment to interventions (eg, trial participants, care providers, outcome assessors, data analysts), and how	_____N/A____
	17b	If blinded, circumstances under which unblinding is permissible, and procedure for revealing a participant's allocated intervention during the trial	_____N/A_____

Methods: Data collection, management, and analysis

Data collection methods	18a	Plans for assessment and collection of outcome, baseline, and other trial data, including any related processes to promote data quality (eg, duplicate measurements, training of assessors) and a description of study instruments (eg, questionnaires, laboratory tests) along with their reliability and validity, if known. Reference to where data collection forms can be found, if not in the protocol	_____9-11____
	18b	Plans to promote participant retention and complete follow-up, including list of any outcome data to be collected for participants who discontinue or deviate from intervention protocols	____12____

Data management	19	Plans for data entry, coding, security, and storage, including any related processes to promote data quality (eg, double data entry; range checks for data values). Reference to where details of data management procedures can be found, if not in the protocol	___14____
Statistical methods	20a	Statistical methods for analysing primary and secondary outcomes. Reference to where other details of the statistical analysis plan can be found, if not in the protocol	_____13____
	20b	Methods for any additional analyses (eg, subgroup and adjusted analyses)	_____13____
	20c	Definition of analysis population relating to protocol non-adherence (eg, as randomised analysis), and any statistical methods to handle missing data (eg, multiple imputation)	_____13____

Methods: Monitoring

Data monitoring	21a	Composition of data monitoring committee (DMC); summary of its role and reporting structure; statement of whether it is independent from the sponsor and competing interests; and reference to where further details about its charter can be found, if not in the protocol. Alternatively, an explanation of why a DMC is not needed	_____14_____
	21b	Description of any interim analyses and stopping guidelines, including who will have access to these interim results and make the final decision to terminate the trial	_____14______
Harms	22	Plans for collecting, assessing, reporting, and managing solicited and spontaneously reported adverse events and other unintended effects of trial interventions or trial conduct	____10-11____
Auditing	23	Frequency and procedures for auditing trial conduct, if any, and whether the process will be independent from investigators and the sponsor	_____14______

Ethics and dissemination

For peer review only - http://bmjopen.bmj.com/site/about/guidelines.xhtml

Research ethics approval	24	Plans for seeking research ethics committee/institutional review board (REC/IRB) approval	_____14______
Protocol amendments	25	Plans for communicating important protocol modifications (eg, changes to eligibility criteria, outcomes, analyses) to relevant parties (eg, investigators, REC/IRBs, trial participants, trial registries, journals, regulators)	_____14______
Consent or assent	26a	Who will obtain informed consent or assent from potential trial participants or authorised surrogates, and how (see Item 32)	_____7-8_____
	26b	Additional consent provisions for collection and use of participant data and biological specimens in ancillary studies, if applicable	_____N/A_____
Confidentiality	27	How personal information about potential and enrolled participants will be collected, shared, and maintained in order to protect confidentiality before, during, and after the trial	_____14______
Declaration of interests	28	Financial and other competing interests for principal investigators for the overall trial and each study site	_____14______
Access to data	29	Statement of who will have access to the final trial dataset, and disclosure of contractual agreements that limit such access for investigators	_______14_____
Ancillary and post-trial care	30	Provisions, if any, for ancillary and post-trial care, and for compensation to those who suffer harm from trial participation	____N/A_____
Dissemination policy	31a	Plans for investigators and sponsor to communicate trial results to participants, healthcare professionals, the public, and other relevant groups (eg, via publication, reporting in results databases, or other data sharing arrangements), including any publication restrictions	_____14_____
	31b	Authorship eligibility guidelines and any intended use of professional writers	____14______

For peer review only

	31c	Plans, if any, for granting public access to the full protocol, participant-level dataset, and statistical code	______N/A______
**Appendices**			
Informed consent materials	32	Model consent form and other related documentation given to participants and authorised surrogates	__Not supplied__
Biological specimens	33	Plans for collection, laboratory evaluation, and storage of biological specimens for genetic or molecular analysis in the current trial and for future use in ancillary studies, if applicable	______N/A______

For peer review only
