# Supplementary material for: Protocol for a feasibility randomised controlled trial of Screening and Enhanced Risk management for Vascular Event-related Decline in Memory (SERVED Memory)
Source: BMJ Open. 2017 Nov 28;7(11):e017416. doi: 10.1136/bmjopen-2017-017416 (PMC5719289; doi:10.1136/bmjopen-2017-017416)
Supplement: Reviewer comments [file bmjopen-2017-017416.reviewer_comments.pdf]

# PEER REVIEW HISTORY

BMJ Open publishes all reviews undertaken for accepted manuscripts. Reviewers are asked to complete a checklist review form (**http://bmjopen.bmj.com/site/about/resources/checklist.pdf**) and are provided with free text boxes to elaborate on their assessment. These free text comments are reproduced below.

## ARTICLE DETAILS

| | |
|---|---|
| **TITLE (PROVISIONAL)** | Protocol for a Feasibility Randomised Controlled Trial of Screening & Enhanced Risk Management for Vascular Event related Decline in Memory (SERVED Memory) |
| **AUTHORS** | Myint, Phyo; Loke, Yoon; Davison, William; Mattishent, Katharina; Fox, Chris; Fleetcroft, Robert; Turner, David; Shepstone, Lee; Potter, John |

## VERSION 1 – REVIEW

| | |
|---|---|
| **REVIEWER** | Michael Brainin MD Professor<br>Clinical Neurosciences<br>Danube Univeristy Krems, Austria<br>No Competing Interest |
| **REVIEW RETURNED** | 28-May-2017 |

| | |
|---|---|
| **GENERAL COMMENTS** | This Trial described investigates the randomization of subjects as a principle problem and thus lays out a feasibility study. In contrast, and due to the published literature (Matz K et al. 2015, Teuschl Y et al. 2017) the problem is rather the adherence of patients in the study and to keep up the strength of the interventions. The intervention described here does hardly comprise all modfiable risk factors and will foreseeably show no group differences. These are very strong limitations if not reservations and should be considered. |

| | |
|---|---|
| **REVIEWER** | Sarah T Pendlebury<br>Centre for Prevention of Stroke and Dementia, Nuffield Department of Clinical Neurosciences, University of Oxford and Departments of Acute Medicine and Geratology, John Radcliffe Hospital, Oxford |
| **REVIEW RETURNED** | 19-Jul-2017 |

| | |
|---|---|
| **GENERAL COMMENTS** | General comments<br><br>This paper describes the rationale and protocol for a study of the feasibility of identifying patients with impaired cognitive function after TIA/stroke for randomisation into an intervention trial of VRF control vs standard care. Cognitive decline in patients with cerebrovascular disease is important and may prove more tractable than pure neurodegenerative dementia. More detail is required on the existing literature on dementia after stroke and the role of case-mix and VRFs so that the study is better set in context of what is already known.<br>Specific comments |

$EVWUDFW LQWURGXFWLRQ ,W LV VWDW
GHFOLQH HVSHFLDOO\ LQ YDVFXODU GHP
necessarily provide the rationale for a study of VRF management to reduce cognitive decline after TIA/stroke since the evidence supports a role for mid-

noted as limitation and this would be something to consider changing when designing a future definitive trial.

Comment ,QWURGXFWLRQ %DFNJURXQG SDJH :KDW LV WKH HYLGHQ 9D'´" 'DWD IURP SRSXODWLRQ VWXGLHV ng age-specific incidence of &)$6 VKRZ D GHF dementia, particularly in men.

Response: This statement has been removed from the introduction as we are interested in post-stroke dementia, rather than VaD per se.

Comment 5) Introduction, page 4. There are many studies of post-stroke dementia with highly heterogenous reported rates of dementia. The current study should be better set in the context of the totality of the data available from systematic reviews of the area which indicate that case-mix is the most important factor underlying variation in dementia risk.

Response: We have amended the background sections to include reference to the importance of case-mix in relation to reported rates of post-stroke dementia. In consideration of this our cohort, which includes both patients with first-ever stroke/TIA and recurrent events, may shed some light on whether number of risk factors or their control is important in relation to post-stroke cognitive impairment.

Comment ,QWURGXFWLRQ SDJH :KDW LV PHDQW E\ ?D VLJQLIL what proportion of these patients have DOUHDG\ KDG 0&,´ do the authors mean that many patients have pre-stroke cognitive decline?

Response: We meant that some patients with stroke/TIA have cognitive decline prior to their cerebrovascular event. This has been clarified in the introduction.

Comment 7) Introduction, page 4. How does the benefit of early diagnosis relate to strategies to prevent VaD?

Response: Identification of early cognitive decline may provide a window of opportunity to instigate treatment which prevents progression to dementia, though granted there are no confirmed strategies at present. This has been clarified in the rationale for the study.

Comment 8) Introduction, page 4. The section on VRF and cognitive decline should be set in context of what is known about post-stroke dementia. Overall, the association with VRF appears weak particularly for hypertension.

Response: Please see the response to earlier comment (number 1) about VRF and their influence on dementia in relation to the abstract.

Comment 9) Rationale, page 5. See earlier comments: variation in dementia diagnostic method in fact accounts for very little of the heterogeneity between studies whereas methodological differences (eg population vs hospital-based, inclusion/exclusion of previous stroke or prestroke dementia) have substantial effects.

Response: Please see the response above relating to the comment (number 1) on the introduction.

Comment 10) Rationale, page 5, cognitive testing. I assume the authors meant to cite reference Stroke 2012,43:464-9 here since it is in the reference list and concerns the validation of the MoCA in TIA/stroke cohort and the cut-offs vs formal MCI diagnosis.

Response: Thank you for highlighting this. This citation had been omitted in error and this has now been properly included.

Comment 11) Rationale, page 5, rate of cognitive decline. As stated earlier, there is little evidence for a major role for VRF in cognitive decline in the short term after stroke. Also, there have been other recent studies of VRF interventions with or without other lifestyle measures after stroke and these should be mentioned eg Int J Stroke. 2014 Oct;9(7):932-8

Response: Thank you for mentioning this study. At the time the protocol for our study was originally written this had not been published, but it is clearly relevant. Reference to it has been added to the study rationale. A difference in our study is that we are recruiting patients with signs of early cognitive decline.

Comment 12) Rationale, page 5. The authors are correct regarding the need for observational epidemiology in this area but the current study is not really large enough or with long enough follow-up to provide robust estimates.

Response: We acknowledge that the study design is not optimal for providing robust epidemiological data and, whilst we hope to provide some insight on this, it is not the primary purpose of the study. As with the other feasibility aims we hope this would inform the design of a future trial.

Comment 13) Study objectives, page 5. The selection appears to be made on the basis of cognitive deficit but not with formal MCI diagnosis (as the authors appear to allude to in the previous paragraph). It would be better to use a more general term such as cognitive impairment no dementia or similar.

Response: We agree that this needs clarification and the statement in this section on the study objectives has been amended accordingly.

Comment 14) Identification of participants, page 7. Validation studies of MoCA in patients with TIA and stroke indicate that the cut-off of >/=26 may be too high to define normal. Also since risk of future cognitive decline is associated with low cognitive score at baseline (or at later time points in those developing late post-stroke dementia), it is unlikely that patients with MoCA>/=26 will show decline over 1 year FU.

Response: We thank the reviewer for this insightful comment. Using the cut-off of >/=26 increases sensitivity which is appropriate for a screening test. However, altering the cut-off value may be something to consider in the future definitive study in order to maximise the chances of detecting cognitive decline.

Comment 15) Intervention to be measured, page 8. The cited intervention trial in Germany had an 8-year FU period whereas the current study has only 1-year.

Response: We cited this study to provide context that the management of risk factors can improve outcomes in older adults. We acknowledge the difference in length of follow-up and would highlight that our trial is a feasibility trial in that context, seeking to examine the potentially meaningful trends in accelerated decline between control and intervention groups, but not seeking to establish definitive results.

Comment 16) Plasma lipids, page 8. Dementia occurring in the context of TIA/stroke may be VaD or mixed and may occur in a more insidious manner ie it is not always stepwise if there is a large AD or small vessel disease component.

Response: We acknowledge that the progression of post-stroke dementia can be variable depending on the underlying pathology and have amended this section of the manuscript accordingly. In addition we accept that lowering cholesterol may only be beneficial in terms of a VaD component. However, statins have additional pleiotropic effects which may result in alternative benefits.

Comment 17) Allocation into study arms, page 9. A score of

Response: The statement in this section regarding MoCA score <17 being consistent with dementia has been referenced. Patients with MoCA score <20 were excluded from the trial and their GP was informed of the MoCA score so that appropriate follow-up could be arranged.

Comment 18) Assessments, page 10. Is demographic information including education collected? This will be needed to establish whether the intervention and control groups are matched at baseline.

Response: Demographic information is being collected. Details of what is being collected have been added to the section on assessments. Some indication of educational level is included in the MoCA, but we are not collecting further details on this. Again, this is something that may need to be considered when designing any future trials.

Comment 19) Outcome measures, page 11. Will recurrent stroke be included in the outcome measures? This may drive dementia differences between the control and intervention groups if differences are seen.

Response: Recurrent stroke will be recorded as part of the collection of adverse events. This has been clarified in the section on assessments.

**Reviewer: 3**
Reviewer Name: Masafumi Ihara
Institution and Country: Director, Department of Neurology, National Cerebral and Cardiovascular Center, Japan
Competing Interests: None declared

Comment: Dr. Myint et al. designed a clinical study to assess whether patients with early cognitive decline after stroke/transient ischaemic attack (TIA) can be easily identified and whether target driven vascular risk factor (VRF) management can prevent progression to dementia. The authors are going to recruit 100 patients with MCI post-stroke/TIA (M0CA 20-25), who will be randomized to intervention (N=50) or control (N=50) groups and assessed for their outcomes at 12 months. A further 100 patients with normal cognition (MoCA>=26) will be also recruited. This study is very meaningful based on the fact that post-stroke dementia is potentially preventable and treatable. I have only minor comments.

Response: We thank the reviewer for their acknowledgement of the importance of the study.

The following concerns if addressed would improve this manuscript.

Comment 1) Figure 1 and Figure 2 seem to be redundant. Can you combine these two figures?

Response: Figures 2 and 3 have been removed as figure 1 provides sufficient information.

Comment 2) Some of the letters do not fit in the frames in Figure 2.

Response: Please see the response to comment 1.

Comment 3) I could not find figure legends for Figure 1-3.

Response: Legends have been added to figures/tables.

Comment 4) Table 1 & 2 should be combined.

Response: Tables 1 and 2 have been combined.

Comment 5) Page 7, line 11; what is PIS?

Response: Thank you for pointing out that this abbreviation had not been defined. This has been amended in the section about assessing capacity and obtaining consent.

Comment 6) Methods for statistical analysis should be added.

Response: Details of the analysis plan are contained within the protocol.

Comment 7) I would like to recommend citing the following paper:
The following paper https://www.ncbi.nlm.nih.gov/pubmed/25105544

Response: We hope that our revisions have improved the manuscript and we look forward to hearing from you again once the revised submission has been reviewed.

**VERSION 2 – REVIEW**

| | |
|---|---|
| **REVIEWER** | Dr ST Pendlebury<br>NIHR Oxford Biomedical Research Centre, John Radcliffe Hospital, Oxford and the Centre for Prevention of Stroke and Dementia, Nuffield Department of Clinical Neurosciences, University of Oxford. |
| **REVIEW RETURNED** | 25-Sep-2017 |

| | |
|---|---|
| **GENERAL COMMENTS** | The authors have responded appropriately to the reviewers' comments |

| | |
|---|---|
| **REVIEWER** | Masafumi Ihara<br>National Cerebral and Cardiovascular Center |
| **REVIEW RETURNED** | 03-Oct-2017 |

| | |
|---|---|
| **GENERAL COMMENTS** | The authors sufficiently revised the manuscript. |
